# FGF18 alleviates sepsis-induced acute lung injury by inhibiting the NF-κB pathway

**DOI:** 10.1186/s12931-024-02733-1

**Published:** 2024-02-28

**Authors:** Zhenyu Hu, Jindan Dai, Tianpeng Xu, Hui Chen, Guoxiu Shen, Jie Zhou, Hongfang Ma, Yang Wang, Litai Jin

**Affiliations:** 1 School of Pharmaceutical Sciences, Wenzhou Medical University, Wenzhou, China; 2 School of Basic Medical Sciences, Wenzhou Medical University, Wenzhou, China

**Keywords:** Fibroblast growth factor 18, Acute lung injury, Inflammation, NF-κB

## Abstract

**Background:**

Acute lung injury (ALI) is a devastating clinical disorder with a high mortality rate, and there is an urgent need for more effective therapies. Fibroblast growth factor 18 (FGF18) has potent anti-inflammatory properties and therefore has become a focus of research for the treatment of lung injury. However, the precise role of FGF18 in the pathological process of ALI and the underlying mechanisms have not been fully elucidated.

**Methods:**

A mouse model of ALI and human umbilical vein endothelial cells (HUVEC) stimulated with lipopolysaccharide (LPS) was established in vivo and in vitro. AAV-FGF18 and FGF18 proteins were used in C57BL/6J mice and HUVEC, respectively. Vascular cell adhesion molecule-1 (VCAM-1), intercellular adhesion molecule-1 (ICAM-1), interleukin-6 (IL-6), tumor necrosis factor-alpha (TNF-α), and p65 protein levels were determined by western blotting or immunofluorescent staining. Afterward, related inhibitors were used to explore the potential mechanism by which FGF18 relieves inflammation.

**Results:**

In this study, we found that FGF18 was significantly upregulated in LPS-induced ALI mouse lung tissues and LPS-stimulated HUVECs. Furthermore, our studies demonstrated that overexpressing FGF18 in the lung or HUVEC could significantly alleviate LPS-induced lung injury and inhibit vascular leakage.

**Conclusions:**

Mechanically, FGF18 treatment dramatically inhibited the NF-κB signaling pathway both in vivo and in vitro. In conclusion, these results indicate that FGF18 attenuates lung injury, at least partially, via the NF-κB signaling pathway and therefore may be a potential therapeutic target for ALI.

**Supplementary Information:**

The online version contains supplementary material available at 10.1186/s12931-024-02733-1.

## Introduction

Acute lung injury (ALI) is a life-threatening medical condition associated with high morbidity and mortality rates [1], which always characterized by widespread lung inflammation and loss of endothelial integrity [2, 3]. Unfortunately, there is currently no effective pharmacological treatment for ALI. Lipopolysaccharide (LPS) is commonly used to induce an immune response in endothelial cells and is widely employed in mouse models to induce ALI [4, 5]. The development of acute pulmonary inflammation is closely tied to the activation of the mitogen-activated protein kinase (MAPK) and nuclear transcription factor-kappa B (NF-κB) signaling pathways [6, 7]. Abnormal NF-κB activation is a defining feature of ALI [8, 9], while MAPK kinases (p38, JNK, ERK1/2) play crucial roles in cellular responses to inflammation [10, 11].

The fibroblast growth factor family consists of 23 members, many of which have the ability to reduce cellular inflammation [12, 13]. FGF18, in particular, plays a vital role in skeletal growth and limb development [14, 15]. Several studies have shown that FGF18 can effectively alleviate cellular inflammation. For instance, X-G Li et al. discovered that FGF18 can reduce inflammation levels in alveolar epithelial cells II caused by hyperoxia [16] and K-Q Sun et al. found that FGF18 is involved in the protective effects of vasoactive intestinal peptide on inflammation induced nucleus pulposus cell degeneration [17]. In addition, FGF18 participates in alveolar development during late embryonic lung development and promotes elastogenesis of pulmonary myofibroblasts [18]. However, the role of FGF18 in the lung, particularly in response to ALI, has not been fully elucidated.

In our current study, we investigated the expression of FGF18 in LPS-treated mice compared to sham controls, and found that FGF18 was upregulated in LPS-treated mice. Furthermore, we examined the therapeutic potential of FGF18 and revealed that FGF18 can alleviate cell inflammation by restraining p65 activation. Moreover, we explored the effects of FGF18 on the expression of pro-inflammatory mediators and cell adhesion molecules after LPS stimulation. The results of this study are of significant value for the treatment of ALI in humans.

## Methods

### Materials

FGF18 (Z03011) was purchased from GenScript (New Jersey USA) and was stored in powder form at − 80℃. JSH-23 (CAS: 749886-87-1), SP600125 (CAS: 129-56-6) and SB203580 (CAS: 152121-47-6) were purchased from Selleck Chemicals (Houston USA). U0126 (CAS: HY-12,031) and Bay11-7082 (CAS: 19542-67-7) was purchased from Med Chem Express (New Jersey USA). Si-*Erk* (sc-29,307) was purchased from Santa Cruz (Texas USA). LPS (L8880) and Tamoxifen (IT0030) were purchased from Solarbio (Beijing CHINA).

### Cell culture experiments

HUVECs were purchased from Lonza and cultured in DMEM (Gibco, C11995500BT) supplemented with 10% FBS (Gibco, 16000-044) and 1% penicillin/streptomycin in an incubator containing 95% air and 5% CO_2_ at 37℃. Before starting the experimental procedures, the medium was removed and replaced with phenol red-free low-glucose DMEM (Gibco, 11,054,020) supplemented with 1% FBS for 12 h, then HUVECs were treated with LPS (100 ng/mL) in the presence of FGF18 (10 ng/mL) for 6 h. HUVECs were treated with SP600125 (10 µM) / SB203580 (10 µM) / si-*Erk* in the presence of LPS and FGF18. Then cells were harvested for western blotting analysis and immunofluorescent staining.

### Animals

Male C57BL/6J mice were purchased from Shanghai SLAC Laboratory Animal Co. Ltd. FGF18 (1 × 10^11^) overexpression vector (AAV9-FGF18) and control vector (AAV9-LacZ) were intratracheally delivered to the mice and stably expressed for 2 weeks. Then to induce ALI, LPS (5 mL/kg) was injected into the mice by intratracheal instillation under isoflurane anesthesia and sacrificed 12 h later. AAV-LacZ and AAV-FGF18 (contract number: HYKY-230,510,005-YAAV) were constructed and purchased from OBiO Technology (Shanghai) Corp., Ltd.

FGF18-CreER^T2^ mice (C57BL/6J background) were bred with ROSA26-td Tomato mice to generate FGF18-CreER^T2^-ROSA26-td Tomato mice. For FGF18-CreER^T2^-ROSA26-td Tomato mice, tamoxifen (Santa Cruz Biotechnology, sc-208,414) was administered at the dose of 75 mg/kg/day for 5 consecutive days by intraperitoneal (i.p.) injection. After the injection, mice were kept for a 14-day waiting period to get the efficient gene knockout. After 12 h of the LPS challenge, the FGF18-CreER^T2^-ROSA26-td Tomato mice were euthanized and lung tissue were taking out. All experimental procedures for animal studies were carried out conforming to the guide for the care and use of laboratory animals and were approved by the Animal Policy and Welfare Committee of Wenzhou Medical University, Wenzhou, Zhejiang Province, CHINA.

The FGF18 heterozygous (*FGF18*^*+/−*^) mice on C57BL/6J background were a generous gift from Professor Shen of Wenzhou University. *FGF18*^*+/−*^ mice primer sequences are shown in Supplementary Table 1. All animals were kept in a standard laboratory condition of 12 h light/darkness cycles, with water and food available ad libitum. All animals received humane care according to the criteria outlined in the Guide for the Care and Use of Laboratory Animals.

### RNA extraction and real-time quantitative RT-PCR

Total RNA from lung tissues or HUVECs was extracted by Trizol reagent (Takara Bro Inc, 9108) according to the manufacturer’s instructions. 1 ng total RNA was reverse transcribed to generate cDNA by the Hiscript ® III Reverse Transcriptase kit (Vazyme). The cDNA was then subjected to RT-PCR analysis. The *GAPDH* gene was used as a control to target gene expression. The specific primer sequences are shown in Supplementary Table 1.

### Nuclear/cytoplasmic fractionation

HUVECs were subjected to nuclear and cytosolic fractionation using the Nuclear/Cytosol Fractionation Kit (Abcam, ab289882), following the protocol recommended by the manufacturer.

### Western blotting analysis

The supernatants from lung tissues or cells were extracted by lysis buffer and protein concentration were determined using Pierce BCA Protein Assay Reagent (Thermo Fisher Scientific, 23,228). 30 µg protein extracts were loaded and separated by SDS-PAGE and transferred to PVDF membranes (Merck Millipore, IPVH00010). Membranes were blocked with 5% bovine serum albumin in Tris-buffered saline containing 0.1% Tween 20 (TBST) and incubated with specific primary antibodies overnight at 4℃. Primary antibodies were as follows: FGF18 (Santa Cruz, sc-393,471), p65 (HUABIO, ET1603-12), p-p65 (HUABIO, ET1604-27), IκBα (Proteintech, 10268-1-AP), p-IκBα (CST, 2859 S), ICAM-1 (Proteintech, 16174-1-AP), VCAM-1 (Affinity, DF6082), IL-6 (HUABIO, HA601051), TNF-α (HUABIO, ER65189), β-actin (ABclonal, AC026), GAPDH (HUABIO, ET1601-4), Lamin B (HUABIO, ET1606-27). Membranes incubated with either goat-anti-mouse HRP (Abcam, ab6789) or goat-anti-rabbit HRP (Abcam, ab6721) for 2 h at room temperature. Proteins were visualized using Amersham Imager 680 (GE Healthcare) system. The expression of specific antigens was quantified using Image J software.

### RNA interference

HUVECs were transfected with FGF18 small interfering RNA (si-*FGF18*; Santa Cruz, sc-39,478) or si RNA scrambled (negative) control (si scramble; Santa Cruz, sc-37,007) by using Lipofectamine 2000 (Thermo Fisher Scientific, 11,668,019) for 6 h in Opti-MEM (Thermo Fisher Scientific, 31985-070). After transfected with si-*FGF18*, HUVECs were treated with LPS in the presence or absence FGF18 for 6 h.

### Immunofluorescent staining

For lung tissues, frozen lung sections were used. For HUVECs, cells cultured on glass coverslips were fixed in 4% paraformaldehyde for 20 min. Frozen lung sections or cells permeabilized in PBS with 0.5% Triton X-100 for 15 min at room temperature. Blocked as above, and then incubated with anti-FGF18 antibody (Santa Cruz, sc-393,471), anti-p65 antibody (Santa Cruz, sc-8008), anti-ICAM-1 antibody (Affinity, DF7413), anti-VCAM-1 antibody (Affinity, DF6082), anti-CD34 (Abcam, ab81289), anti-Aquaporin 5 (Abcam, ab78486), anti-Prosurfactant Protein C (Abcam, ab211326), anti-CD34 (Abcam, ab81289), anti-VE-cadherin (Abcam, ab33168), anti-F4/80 (CST, 30,325 S), anti-CD3 (Proteintech, 17617-1-AP) at 4℃ overnight. And then it was incubated with a secondary antibody of Alexa Fluor 488-conjugated anti-mouse IgG (1:200) (Abcam, ab150113) or Alexa Fluor 647-conjugated anti-rabbit IgG secondary antibody (1:200) (Abcam, ab150075). Finally, the nuclei were stained with DAPI. Images were captured with a Nikon C2si Confocal microscope (Nikon, Japan).

### Statistical analysis

Data were expressed as the means ± SEM. Differences of samples were performed using an unpaired two-tailed t-test or analysis of variance (ANOVA). Statistical analysis between multiple groups was performed by one-way ANOVA. *P* < 0.05 was considered statistically significant. Statistical analysis was performed by GraphPad Prism 9.4.1.

## Results

### FGF18 expression is increased in ALI mice and LPS-treated HUVECs

**Fig. 1:**
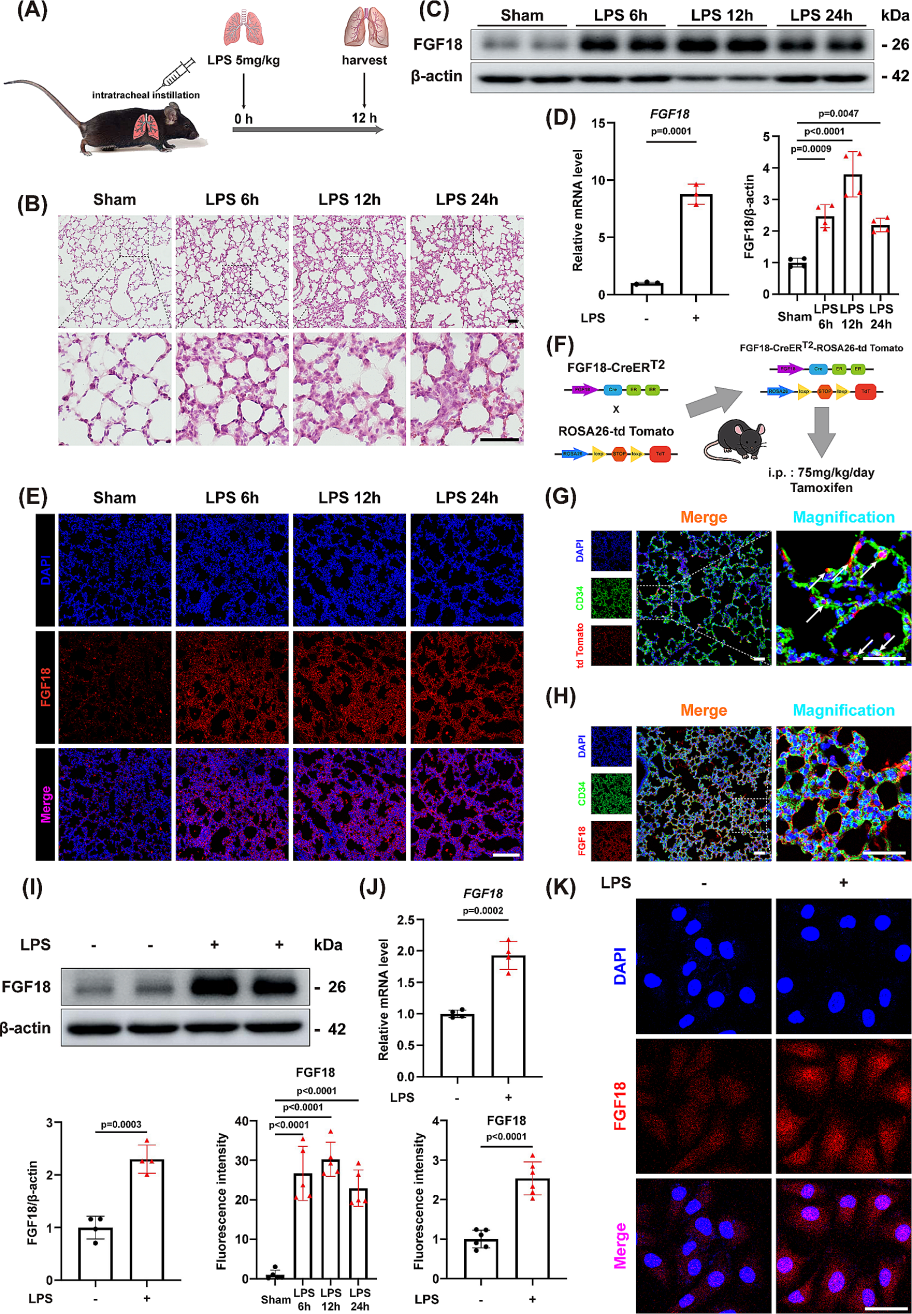
FGF18 expression is increased in ALI mice and LPS-treated HUVECs. **(A)** Schematic diagram demonstrates the animal experiment design. **(B)** Hematoxylin–eosin staining in lung tissues of C57BL/6J mice after PBS or LPS injection for 6 h, 12 h, and 24 h. (*n* = 5 per group, Scale bar = 50 μm). **(C)** Western blotting was performed and quantitatively analyzed to determine the protein levels of FGF18 in the lungs from LPS and PBS-treated controls. (*n* = 4 per group). **(D)** qRT-PCR analysis of the mRNA levels of FGF18 in the lungs of ALI or sham. (*n* = 3 per group). **(E)** Representative immunofluorescent staining analysis of FGF18 proteins in the lung tissues from ALI and sham. (*n* = 5 per group, Scale bar = 150 μm) **(F)** FGF18-CreERT2-ROSA26-td Tomato mice were intraperitoneally injected with 75 mg/kg of tamoxifen dissolved in corn oil for 2 weeks, followed by LPS tracheal infusion. **(G)** Immunofluorescent staining of CD34 (green), td Tomato (red), and DAPI (blue) in FGF18-CreERT2-ROSA26-td Tomato mice were detected. (Scale bar = 50 μm). **(H)** Immunofluorescent staining of CD34 (green), FGF18 (red), and DAPI (blue) in C57BL/6J mice were detected. (Scale bar = 50 μm). **(I)** Western blotting was performed and quantitatively analyzed to determine the protein levels of FGF18 in HUVECs. (*n* = 4 per group). **(J)** qRT-PCR analysis of the mRNA levels of FGF18 in HUVECs. (*n* = 4 per group). **(K)** Immunofluorescent staining of FGF18 (red) and DAPI (blue) in HUVECs were detected. (Scale bar = 50 μm)

As mice treated with 5 mg/kg of LPS for 12 h significantly showed collapsed alveolar structure and infiltration of inflammatory cells. Compared with the control mice, the 12-hour LPS stimulation group showed significant lung injury, therefore this treatment was used in subsequent in vivo experiments (Fig. 1A, B). To determine the involvement of FGF18 in intratracheal LPS-induced ALI in mice, we examined its expression in the lungs of mice intratracheally injected with LPS for 6 h, 12 h, and 24 h. Western blotting and quantitative RT-PCR showed a marked increase in FGF18 expression in the ALI mice at both the mRNA and protein levels, respectively (Fig. 1C, D). In addition, immunofluorescent staining confirmed the significant upregulation of FGF18 expression in lung tissues (Fig. 1E). To further explore the function of FGF18 in ALI mice, ROSA26-td Tomato mice were bred with FGF18-Cre mice to generate FGF18-CreER^T2^-ROSA26-td Tomato mice (Fig. 1F). Interestingly, we observed that CD34, a marker of endothelial cells, could be co-located with cells secreting FGF18 protein (Fig. 1G). Moreover, the co-localization of FGF18 with other cell markers in C57BL/6J mice with ALI were also detected. We found that FGF18 was co-localized with endothelial cells, epithelial cells and macrophages etc. (Fig. 1H, Supplementary Fig. 5A), and in the present study we focused on the endothelial cell. As for the role of FGF18 in other cells, further studies are needed. The in vitro results are consistent with the findings from quantitative RT-PCR, western blotting, and immunofluorescence analysis, which showed that FGF18 was upregulated at both mRNA and protein levels in LPS-treated HUVECs (Fig. 1I-K). Thus, these findings suggest a potential correlation between FGF18 and ALI, as FGF18 expression is consistently increased in the ALI.

### FGF18 alleviates lung injury in the ALI mouse model

**Fig. 2:**
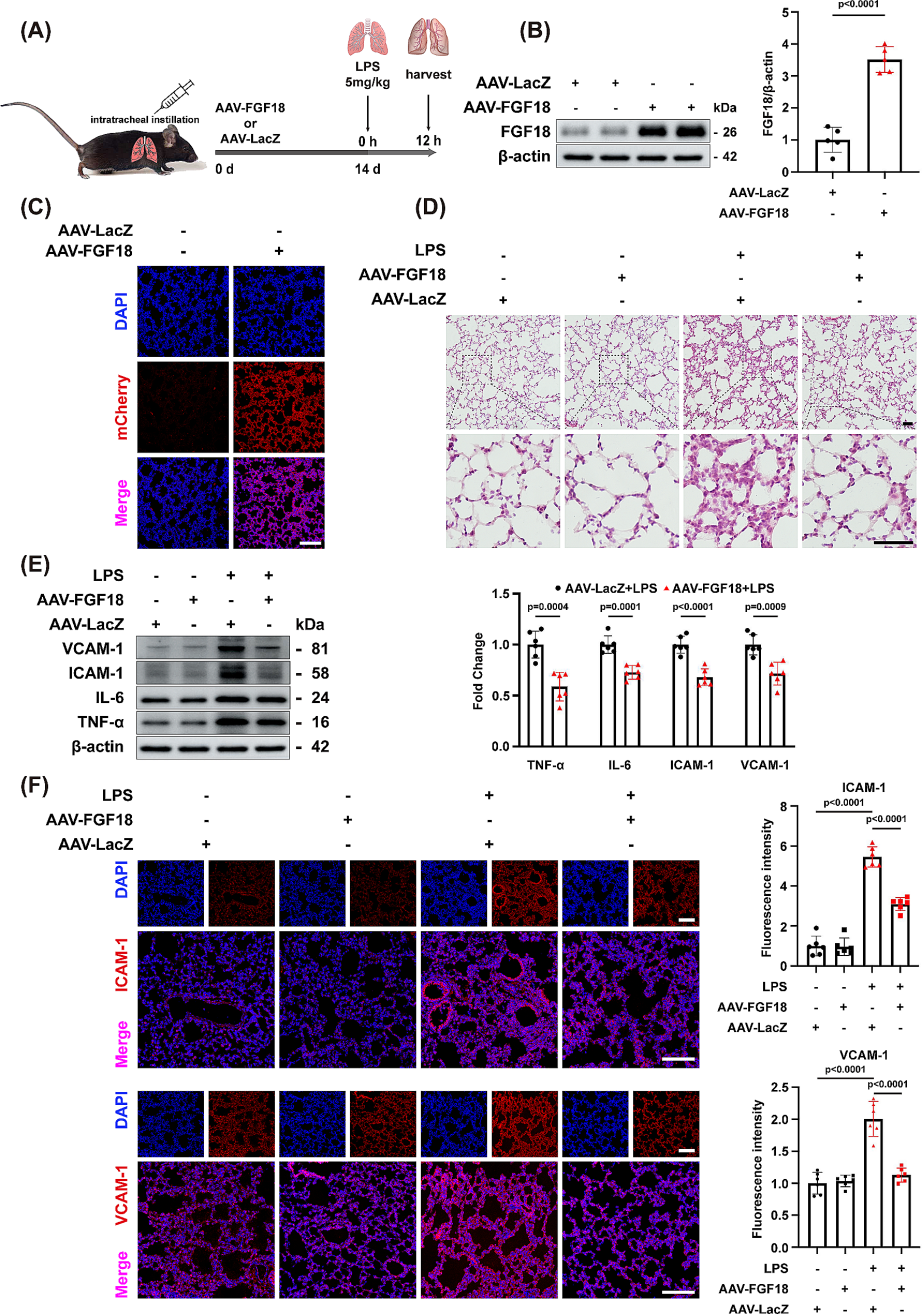
FGF18 alleviates lung injury in the ALI mouse model. **(A)** Schematic diagram demonstrates the animal experiment design. **(B)** Western blotting was performed and quantitatively analyzed to determine the protein levels of FGF18 in the lungs from AAV-FGF18 and AAV-LacZ-treated controls. (*n* = 5 per group). **(C)** Immunofluorescent staining of FGF18 (red) and DAPI (blue) in AAV-FGF18 or AAV-LacZ-treated mice were detected. (Scale bar = 150 μm). **(D)** HE staining in lung tissues of AAV-FGF18 and AAV-LacZ-treated mice after PBS or LPS injection for 12 h. (*n* = 6 per group, Scale bar = 50 μm). **(E)** Western blotting was performed and quantitatively analyzed to determine the protein levels of VCAM-1, ICAM-1, IL-6, and TNF-α in the lungs from AAV-FGF18 and AAV-LacZ-treated controls. (*n* = 6 per group). **(F)** Immunofluorescent staining of ICAM-1/VCAM-1 (red) and DAPI (blue) in AAV-FGF18 and AAV-LacZ-treated mice were detected. (*n* = 6 per group, Scale bar = 150 μm)

FGF18 has been reported to play a crucial role in cellular inflammation. However, its function in ALI remains unclear. In order to explore this, we conducted a study using mice that were genetically modified to specifically overexpress FGF18 in the lung through the use of adeno-associated virus (AAV) 9-FGF18. For comparison, control mice were injected with an equal number of AAV-LacZ particles (Fig. 2A). After a period of 2 weeks, during which stable expression of FGF18 protein was achieved in vivo, the overexpression was confirmed through western blotting analysis (Fig. 2B). The transfection efficiency of AAV was determined using immunofluorescence analysis (Fig. 2C). As anticipated, hematoxylin-eosin (HE) staining revealed that the overexpression of FGF18 led to an improvement in lung injury, as indicated by reduced infiltration of inflammatory cells compared to the control ALI mice (Fig. 2D).

To further investigate the relationship between FGF18 and inflammation, lung sections were subjected to western blotting and immunofluorescent staining. As expected, western blotting showed a decrease in the protein levels of VCAM-1, ICAM-1, IL-6, and TNF-α after treatment with FGF18 (Fig. 2E). Additionally, fewer cell adhesion molecules and an increase in VE-cadherin were observed in mice treated with AAV-FGF18 (Fig. 2F, Supplementary Fig. 2C). These findings collectively suggest that FGF18 effectively ameliorated lung injury in mice with LPS-induced ALI.

### FGF18 contributes to the repair of HUVECs

**Fig. 3:**
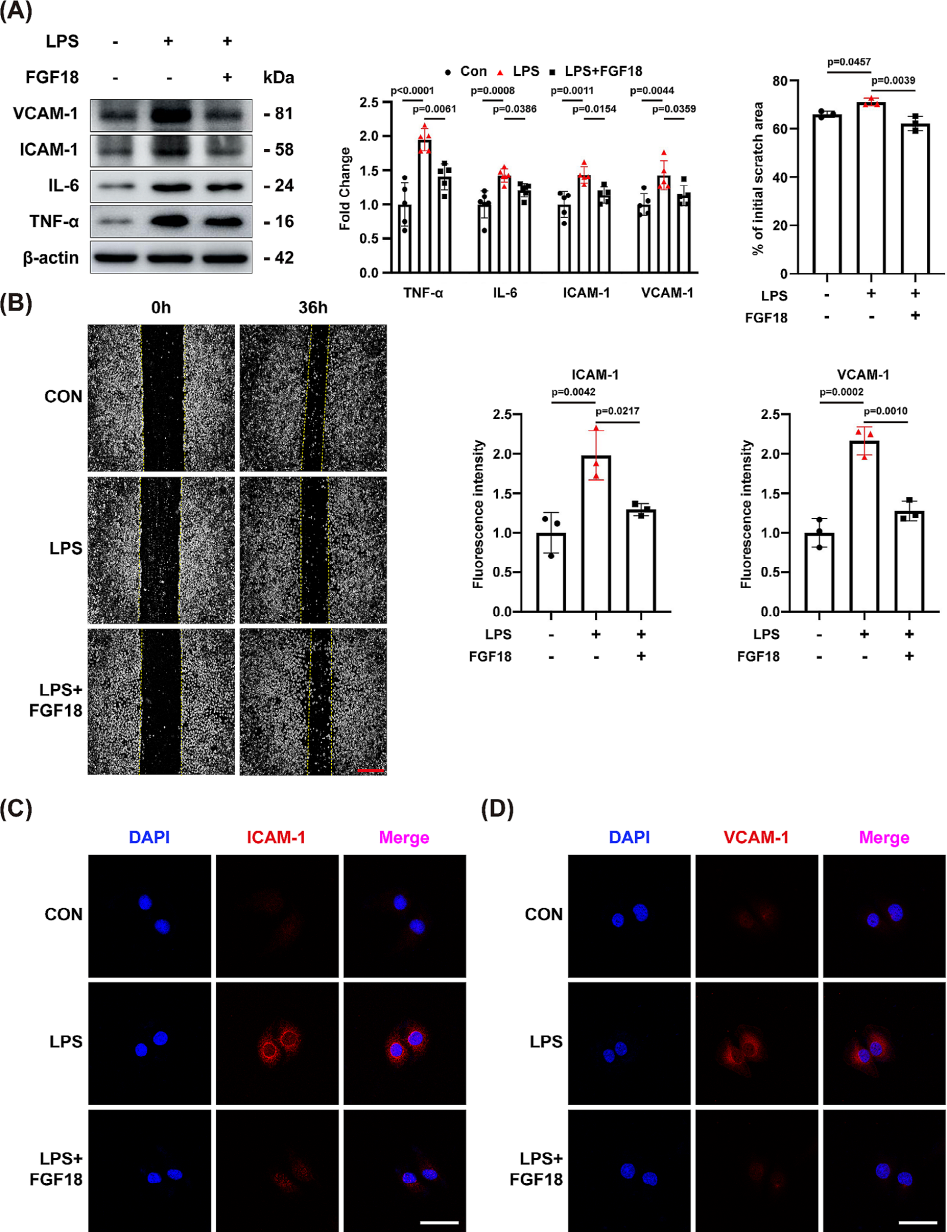
FGF18 contributes to the repair of HUVECs. **(A)** HUVECs were subjected to western blotting analysis. The expression of VCAM-1, ICAM-1, IL-6, and TNF-α was detected. (*n* = 5 per group). **(B)** In vitro, scratch assay showing the changes in migration potential of HUVECs stimulated with LPS in the presence of FGF18. (*n* = 3 per group, Scale bar = 25 μm). **(C)** Immunofluorescent staining of ICAM-1 (red) and DAPI (blue) in HUVECs were detected. (*n* = 3 per group, Scale bar = 150 μm). **(D)** Immunofluorescent staining of VCAM-1 (red) and DAPI (blue) in HUVECs were detected. (*n* = 3 per group, Scale bar = 150 μm)

Impaired pulmonary vascular endothelial barrier function is a typical pathological feature of ALI. To investigate the effect of FGF18 on endothelial impairment in vitro, we conducted a series of experiments. Firstly, we determined several time points for LPS treatment and found that the damage was most severe after 6 h (Supplementary Fig. 1A-F, 1I). Then, we treated HUVECs with human recombinant FGF18 in the presence of LPS, and found that treatment with 10 ng/mL FGF18 significantly relived the injury of HUVECs contributed by LPS. Thus, we used this concentration for subsequent in vitro experiments (Supplementary Fig. 1J). Next, western blotting analysis revealed that FGF18 markedly reduced the LPS-induced expression of proteins, such as VCAM-1, ICAM-1, IL-6, and TNF-α, which are the important biomarkers of HUVEC damage (Fig. 3A). These findings were consistent with the immunofluorescent staining results of ICAM-1 and VCAM-1 (Fig. 3C, D). Moreover, it is well known that reduced expression of VE-cadherin is a marker of endothelial cell damage. Thus, also detected the fluorescence intensity change of VE-cadherin (Supplementary Fig. 2A) and found that FGF18 could significantly alleviate the decline of VE-cadherin induced by LPS. Furthermore, the damage of the HUVECs and the mRNA expression of inflammatory factors bearing FGF18 were improved. (Fig. 3B, Supplementary Fig. 6A). Taken together, these results suggest that FGF18 reduces the injury of HUVECs.

### FGF18 deletion exacerbates LPS-induced lung injury

**Fig. 4:**
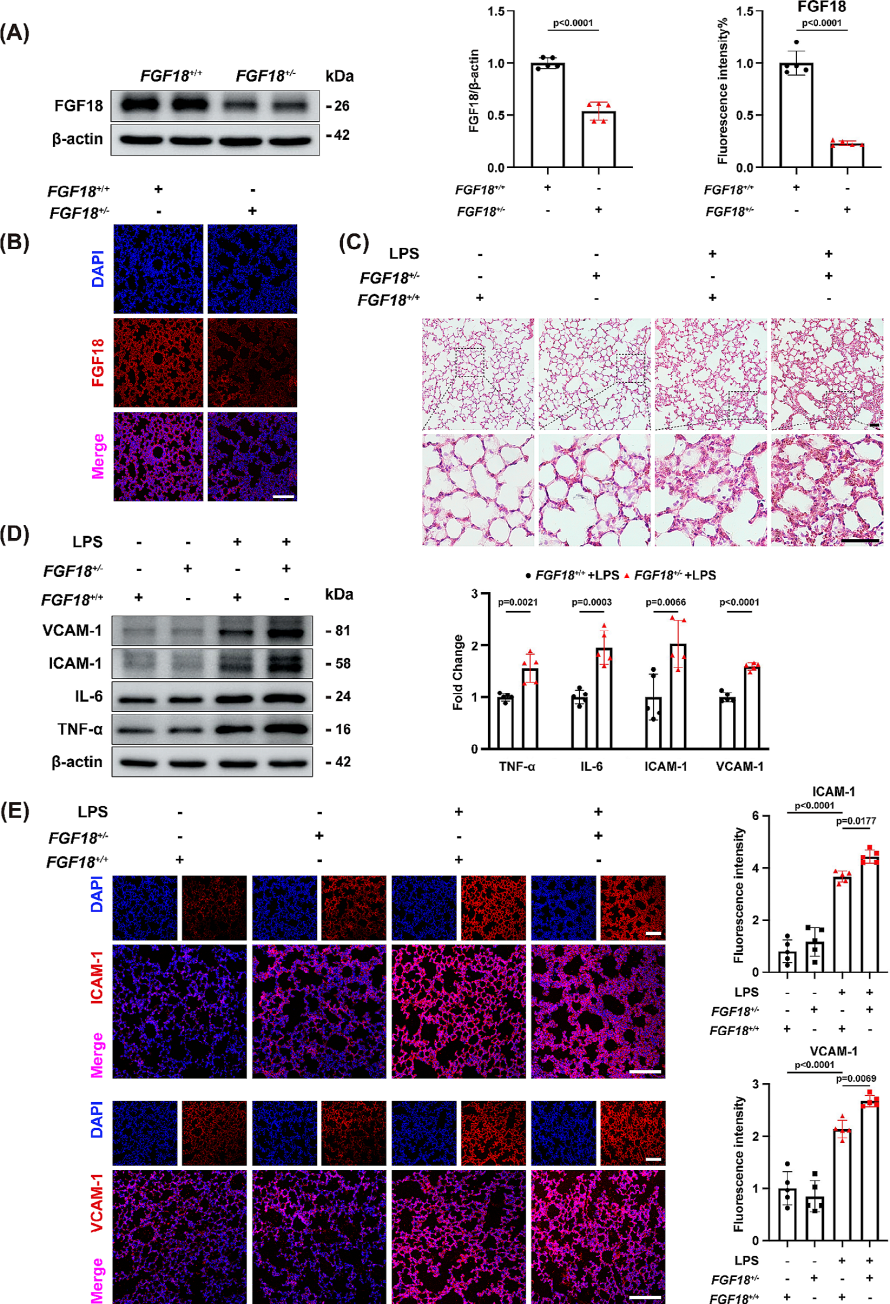
FGF18 deletion exacerbates LPS-induced lung injury. **(A)** *FGF18*^*+/+*^ and *FGF18*^*+/−*^ mice were subjected to western blotting analysis. The expression of FGF18 was detected. (*n* = 5 per group). **(B)** Immunofluorescent staining of FGF18 (red) and DAPI (blue) in *FGF18*^*+/+*^ and *FGF18*^*+/−*^ mice were detected. (*n* = 5 per group, Scale bar = 150 μm). **(C)** HE staining in lung tissues of *FGF18*^*+/+*^ and *FGF18*^*+/−*^ mice after PBS or LPS injection for 12 h (*n* = 5 per group). **(D)** Western blotting was performed and quantitatively analyzed to determine the protein levels of VCAM-1, ICAM-1, IL-6, and TNF-α in the lungs from *FGF18*^*+/+*^ and *FGF18*^*+/−*^ mice in the presence of LPS (*n* = 5 per group). **(E)** Immunofluorescent staining of ICAM-1/VCAM-1 (red) and DAPI (blue) in *FGF18*^*+/+*^ and *FGF18*^*+/−*^ mice were detected. (*n* = 5 per group, Scale bar = 150 μm)

FGF18 primarily signals to mesenchymal tissue during embryonic development in developing lungs, and FGF18 germline KO mice (*FGF18*^−/−^) die shortly after birth. To further explore the regulatory role of FGF18 in LPS-induced lung injury, FGF18 heterozygous mice (*FGF18*^+/−^ mice) were used to evaluate the impact of FGF18 on ALI mice. We measured that FGF18 was significantly decreased in *FGF18*^+/−^ mice compared with WT mice (Fig. 4A, B). RT-PCR further verified the success of FGF18 knockout. (Supplementary Fig. 4B). WT and *FGF18*^+/−^ mice were subject to LPS administration for 12 h and then euthanized to get lung tissues. In addition, we found that the deletion of FGF18 exacerbated lung injury and inflammatory cell recruitment (Fig. 4C). Moreover, FGF18 deficiency significantly aggravated the elevated levels of VCAM-1, ICAM-1, IL-6, and TNF-α, compared to levels in WT LPS-treated mice (Fig. 4D). Meanwhile, more cell adhesion molecules with less VE-cadherin were also observed in *FGF18*^+/−^ mice (Fig. 4E, Supplementary Fig. 3B). Taken together, these results indicate that FGF18 ablation aggravates LPS-induced lung inflammation and injury.

### The knockdown of FGF18 exacerbates LPS-induced HUVEC injury

**Fig. 5:**
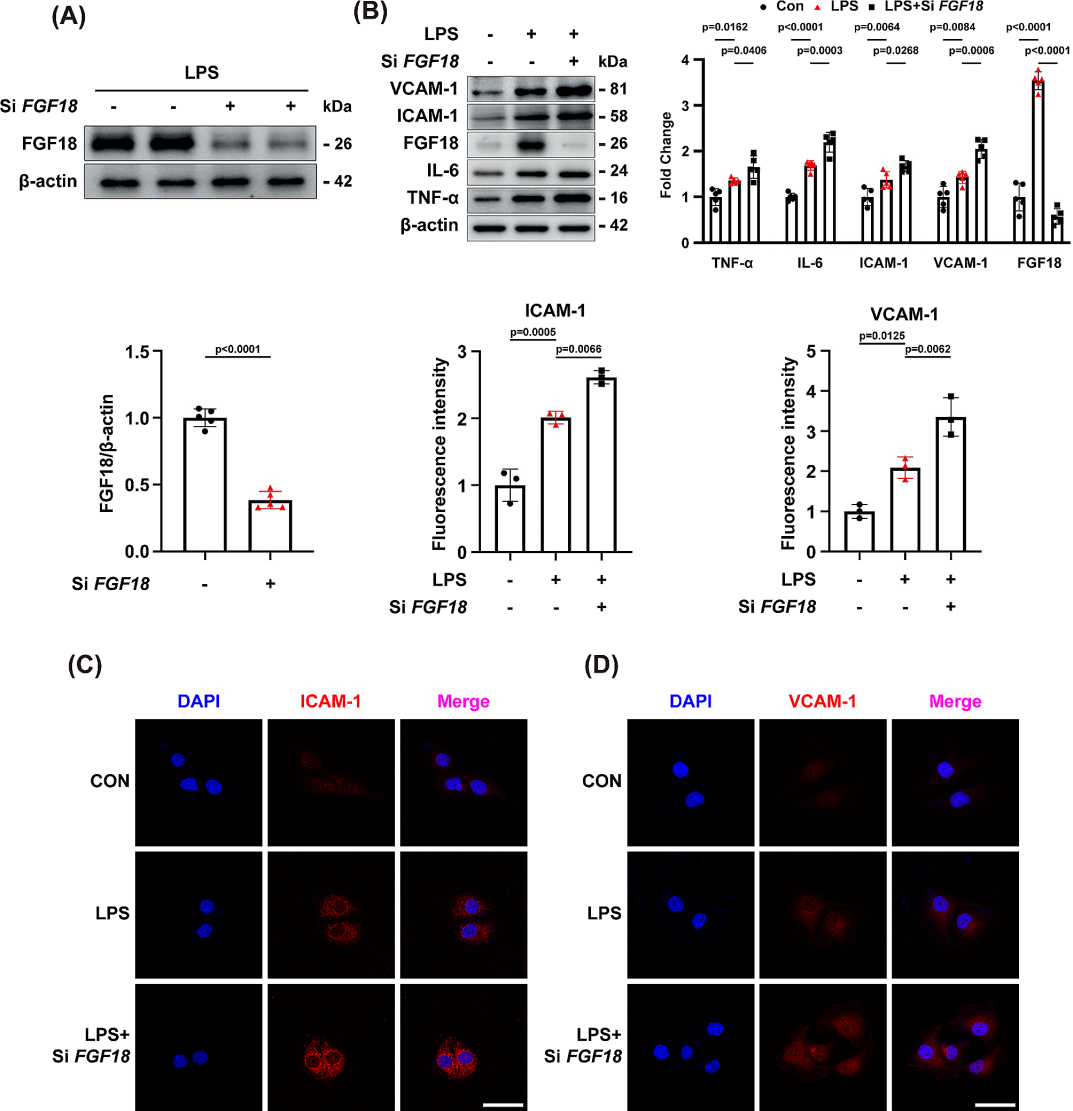
The knockdown of FGF18 exacerbates LPS-induced HUVEC injury. **(A)** Western blotting of FGF18 in HUVECs was detected. (*n* = 5 per group). **(B)** HUVECs were subjected to western blotting analysis. The expression of VCAM-1, ICAM-1, IL-6, and TNF-α were detected. (*n* = 5 per group). **(C)** Immunofluorescent staining of ICAM-1 (red) and DAPI (blue) in HUVECs were detected. (*n* = 3 per group, Scale bar = 50 μm). **(D)** Immunofluorescent staining of VCAM-1 (red) and DAPI (blue) in HUVECs were detected. (*n* = 3 per group, Scale bar = 50 μm)

To further verify the function of FGF18 in LPS treated HUVEC, western blotting analysis showed that FGF18 was significantly decreased after treated with si-*FGF18* (Fig. 5A, Supplementary Fig. 4A). To explore the function of FGF18 in vitro, we found that the protein level of VCAM-1, ICAM-1, IL-6, and TNF-α was further increased in HUVECs transfected with si-*FGF18* (Fig. 5B), and the mRNA level of ICAM-1 and IL-6 was also increased (Supplementary Fig. 1G, H), suggesting that deletion of FGF18 could exacerbate LPS-induced HUVEC damage. Moreover, we found that the elevated level of ICAM-1 and VCAM-1 (Fig. 5C, D) and decreased level of VE-cadherin (Supplementary Fig. 2B) under LPS conditions were further increased or decreased by si-*FGF18* treatment as measured by immunofluorescent staining, respectively. Collectively, these data provide further evidence that knocking down FGF18 could aggravate LPS-induced HUVEC injury.

### FGF18 promotes lung repair and attenuates HUVEC injury by inhibiting the NF-κB pathway

**Fig. 6:**
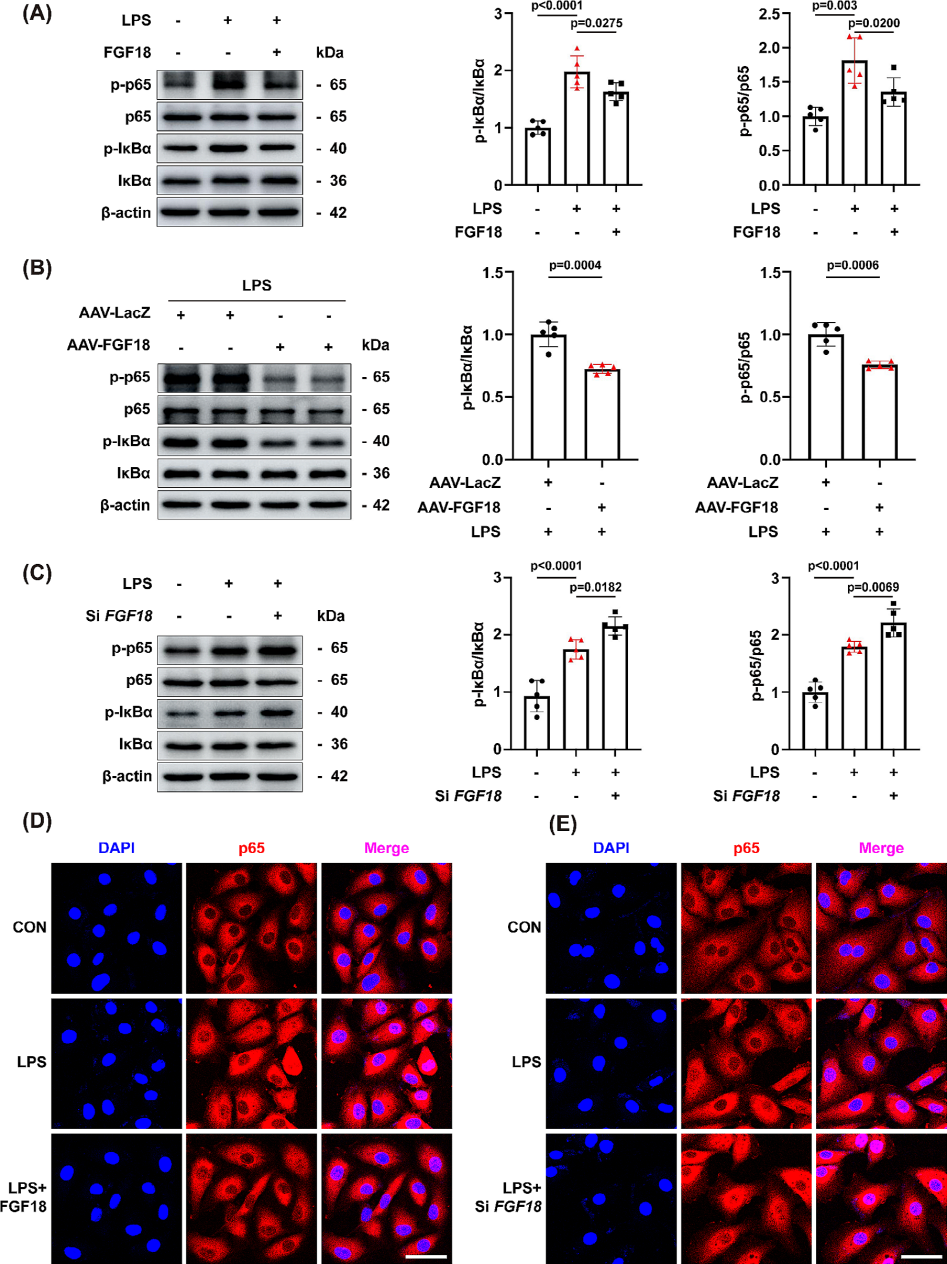
FGF18 promotes lung repair and attenuates HUVEC injury by inhibiting the NF-κB pathway. **(A)** HUVECs were subjected to western blotting analysis. The expression of p-p65, p65, p-IκBα, and IκBα were detected. (*n* = 5 per group). **(B)** C57BL/6J mice were treated with AAV-FGF18 and AAV-LacZ in the presence of LPS and then subjected to western blotting analysis. (*n* = 5 per group). **(C)** HUVECs were subjected to western blotting analysis. The expression of p-p65, p65, p-IκBα, and IκBα were detected. (*n* = 5 per group). **(D)** Immunofluorescent staining of p65 (red) and DAPI (blue) in HUVECs were detected. (*n* = 5 per group, Scale bar = 50 μm). **(E)** Immunofluorescent staining of p65 (red) and DAPI (blue) in HUVECs were detected. (*n* = 5 per group, Scale bar = 50 μm)

**Fig. 7:**
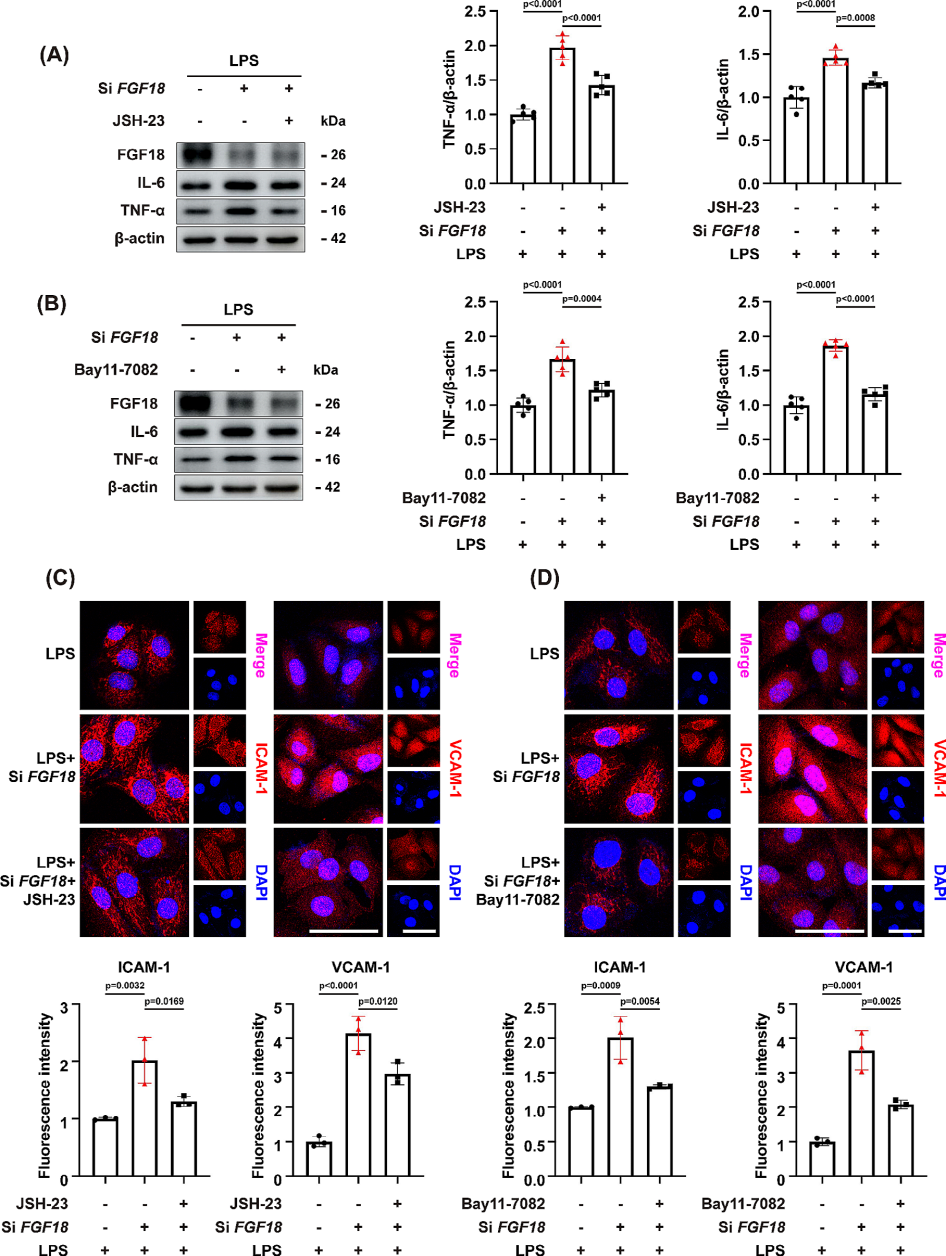
FGF18 promotes HUVECs repair by inhibiting NF-κB p65 activation. **(A)** HUVECs transfected with si-*FGF18* were then treated with JSH-23 (20 µM) for 3 h, HUVECs were subjected to western blotting analysis. The expression of TNF-α and IL-6 were detected. (*n* = 5 per group). **(B)** HUVECs transfected with si-*FGF18* were then treated with Bay11-7082 (10 µM) for 3 h, HUVECs were subjected to western blotting analysis. The expression of TNF-α and IL-6 were detected. (*n* = 5 per group). **(C)** Immunofluorescent staining of ICAM-1/VCAM-1 (red) and DAPI (blue) in HUVECs were detected. (*n* = 3 per group, Scale bar = 50 μm). **(D)** Immunofluorescent staining of ICAM-1/VCAM-1 (red) and DAPI (blue) in HUVECs were detected. (*n* = 3 per group, Scale bar = 50 μm)

LPS treatment caused significant activation of the NF-κB pathway by enhancing the phosphorylation of IκBα and NF-κB p65 in HUVECs and reversed by FGF18 treatment (Fig. 6A). Meanwhile, western blotting analyses revealed that IκBα and NF-κB p65 phosphorylation was significantly decreased in the lungs of AAV-FGF18-treated mice than in the lungs of control mice (Fig. 6B), and higher in *FGF18*^+/−^ mice than in lungs of WT mice (Supplementary Fig. 3A). Additionally, results from in vitro experiments showed that si-*FGF18* treatment increased the phosphorylation of IκBα and NF-κB p65 (Fig. 6C), indicating that FGF18 knockdown enhanced the activation of the NF-κB signaling pathway. Moreover, a nuclear-cytoplasmic separation experiment demonstrated that FGF18 treatment reduced the entry of NF-κB p65 into the nucleus after LPS stimulation, while si-*FGF18* treatment increased the nuclear translocation of NF-κB p65 (Fig. 6D, E). These findings suggest that FGF18 plays a direct protective role against LPS-induced endothelial impairment by inhibiting the activation and nuclear translocation of NF-κB p65. To clarify whether FGF18 protects endothelial cell by inhibiting the NF-κB pathway, we used NF-κB p65 inhibitors JSH-23 and Bay11-7082, which inhibits the expression of p-p65 [19, 20]. The results showed that TNF-α and IL-6 were enriched after si-*FGF18* treatment, and this phenomenon was alleviated by the treatment of JSH-23 and Bay11-7082 (Fig. 7A, B), suggesting that FGF18 could suppress the TNF-α and IL-6 expression via p65. In addition, JSH-23 and Bay11-7082 treatment eliminated the ICAM-1 and VCAM-1 exacerbations induced by si-*FGF18* (Fig. 7C, D).

Overall, the data indicate that FGF18 ameliorates lung injury in LPS-induced ALI by inhibiting the NF-κB signaling pathway and protecting against endothelial impairment.

### FGF18 is consistent with MAPK kinase inhibitors in reducing the activation of NF-κB pathway

**Fig. 8:**
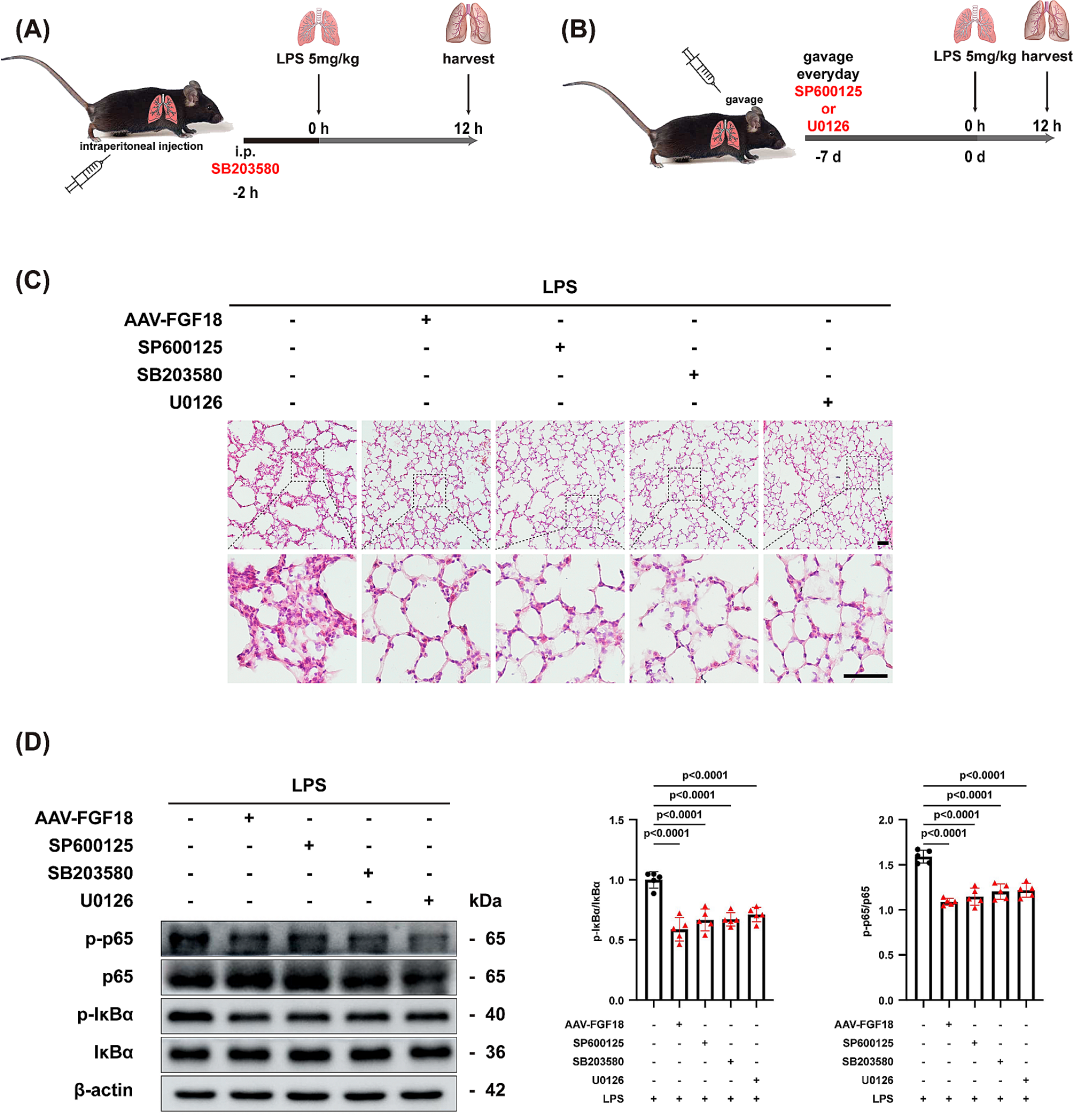
FGF18 inhibits NF-κB pathway activation in ALI mice in line with MAPK kinase inhibitors. **(A, B)** A schematic diagram demonstrates the animal experiment design. **(C)** HE staining in lung tissues of MAPK kinase inhibitors-treated mice in the presence of AAV-FGF18 after LPS injection for 12 h. (*n* = 5 per group, Scale bar = 50 μm). **(D)** C57BL/6J mice were treated with AAV-FGF18 and MAPK kinase inhibitors in the presence of LPS and then subjected to western blotting analysis. (*n* = 5 per group)

MAPK kinases were widely reported to regulate the inflammation-related signaling pathway. Because of the tight association between the MAPK and NF-κB signaling pathways, a large number of studies have shown that MAPK kinase inhibitors could attenuate phosphorylation of NF-κB p65 [19–21]. In the inhibitors group, mice received SB203580 by intraperitoneal injection (Fig. 8A). Moreover, the other two groups of mice underwent gavage of SP600125 and U0126 (Fig. 8B). HE staining revealed that the AAV-FGF18 group and MAPK kinase inhibitors could alleviate lung injury and inflammatory cell aggregation (Fig. 8C). In addition, LPS stimulation enhanced the phosphorylation of IκBα and p65, but this activation was significantly ameliorated by FGF18 and MAPK kinase inhibitors treatment, indicating that FGF18 inhibited LPS-induced activation consistent with MAPK kinase inhibitors in ALI mice (Fig. 8D). Overall, these findings confirm that FGF18 exhibits a comparable protective effect against LPS-induced lung injury as MAPK kinase inhibitors.

The in vitro experiments conducted in HUVECs demonstrated that FGF18, as well as MAPK kinase inhibitors (SP600125 and SB203580), and si-*Erk* could alleviate the phosphorylation of IκBα and p65 (Supplementary Fig. 7A). This indicates that FGF18 and MAPK kinase inhibitors have a similar effect in inhibiting the activation of the NF-κB signaling pathway. Furthermore, western blotting analysis confirmed that pretreatment with FGF18 and the inhibition of MAPK kinases reduced the nuclear localization of IκBα and NF-κB p65 induced by LPS (Supplementary Fig. 7B). This suggests that FGF18 and MAPK kinase inhibitors can prevent the translocation of NF-κB p65 into the nucleus. Immunofluorescent staining further supported these findings by demonstrating that FGF18 and MAPK kinase inhibitors could alleviate the entry of NF-κB p65 into the nucleus (Supplementary Fig. 7C). Intriguingly, the combination of FGF18 and MAPK kinase inhibitors could further inhibit the NF-κB pathway (Supplementary Fig. 8A). Overall, these results provide strong evidence that the therapeutic effect of FGF18 is consistent with that of MAPK kinase inhibitors in inhibiting the NF-κB signaling pathway. This suggests that FGF18 may serve as a novel approach for the treatment of acute lung injury.

## Discussion

ALI and ARDS are the two main causes of acute lung failure, which is characterized by high morbidity and mortality and for which effective therapeutic strategies are lacking. Thus, identifying novel treatments for ALI is urgently needed. Several lines of evidence show that FGFs play a central role in pulmonary inflammation. Dhlamini, Q et al. found that FGF1 alleviates LPS-induced ALI via suppression of inflammation and oxidative stress [22]. Tichelaar JW et al. confirmed that FGF7 improved survival during ALI in adult mouse lungs after short-term expression [23]. Wang Q et al. demonstrated that the anti-inflammatory effect of FGF10 on NF-κB signaling was mediated through the regulation of oxidative stress [24].

It is well-realized that FGF18 is released by interstitial cells and possibly endothelial cells in the lung and is known to drive cell migration [25], especially for endothelial cells [26]. FGF18 transgene induction also enhanced the expression of other genes that may be involved in angiogenesis, including endothelial cell growth and differentiation factor Wnt2 [27]. Interestingly, previous findings identified FGF18 as a likely important player in the control of alveolar angiogenesis [18], an event that is an absolute requirement for alveolarization and is compromised in bronchopulmonary dysplasia. However, the role of FGF18 in the pathological development of ALI has not been reported. In this study, we revealed that FGF18 protects against pulmonary injury by inhibiting the NF-κB pathway both in vivo and in vitro (Fig. 6). FGF18 inhibits nuclear accumulation of NF-κB p65 and thereby, alleviates cellular inflammation and pulmonary repair. These findings provide new clues and ideas for developing potential methods to treat ALI and promote pulmonary repair.

Our previous study demonstrated that FGF18 plays a protective role in the liver, especially in liver fibrosis and hepatic ischemia-reperfusion [28, 29]. In the present study, we found that FGF18 was increased upon LPS stimulation, and FGF18 treatment could decrease the phosphorylation of IκBα, and this effect was also correlated to a parallel decrease in the nuclear translocation of the NF-κB p65 as confirmed by immunofluorescence and western blotting analysis. On the contrary, reducing the expression of FGF18 in vivo and in vitro exacerbates lung injury and endothelial cell damage, respectively (Fig. 4). Our work showed that the elevated p-IκBα and p-p65 expression in LPS-treated HUVECs was largely reversed by FGF18 treatment along with alleviated HUVEC injury (Fig. 6). This is the first time for us to explore the relationship between FGF18 and the NF-κB pathway in the context of acute lung injury.

Double immunofluorescent staining indicated that FGF18 was mainly co-localized with CD34 expression, suggesting that endothelial cells are the main source of FGF18 in mice lungs. Consistently, FGF18 was upregulated in LPS-treated HUVECs (Fig. 1). Lung vascular leakage in response to an unchecked cytokine storm generated by the activation of innate immune cells is a hallmark of sepsis-induced inflammatory injury [30–32]. The vascular leakage in the lungs is the result of endothelial barrier breakdown and the change level of cell adhesion molecules [33, 34]. Endothelial permeability is normally tuned by the interaction of VE-cadherin in endothelial monolayers [35, 36]. Multiple studies have demonstrated that modifications of FGFs are important for the structure of endothelial cells [37]. Furthermore, aberrant NF-κB activation contributes to the development of vascular leakage [38], among other inflammatory disorders, by mediating the transcription of proinflammatory cytokines such as TNF-α, IL-6, and IL-1β, which in turn enhance the inflammatory response. Here, FGF18 pretreatment significantly suppressed LPS-induced phosphorylation of IκBα and NF-κB p65 in mice, consistent with MAPK kinase inhibitors (Fig. 7).

Hyper-phosphorylation of MAPK molecules can lead to the activation of NF-κB and the subsequent production of inflammatory molecules [19–21]. The MAPK and NF-κB pathways have been identified as important targets in LPS-induced ALI. In our study, it was observed that FGF18 significantly attenuated the phosphorylation of NF-κB p65 in a dose-dependent manner, although the specific data was not shown. This finding is consistent with previous studies that have shown MAPK kinase inhibitors can alleviate p65 phosphorylation [39, 40]. Based on these observations, it is reasonable to speculate that FGF18 may affect the NF-κB pathway in a similar manner to MAPK kinase inhibitors. The potential mechanism of FGF18 inhibits p-IκBα and p-p65 may involve several aspects. Firstly, FGF18 may interfere with the activity of kinases involved in the NF-κB signaling pathway, such as the IKK complex (IκB kinase), reduces the phosphorylation of IκBα, thereby inhibiting the activation and intranuclear transfer of NF-κB. Secondly, FGF18 may indirectly affect the NF-κB pathway by affecting the upstream signals of NF-κB activation, such as those of inflammatory factors (TNF-α or IL-1β). Further investigation is still required to fully understand the underlying mechanisms and confirm the specific interactions between FGF18 and the NF-κB pathway.

It appears that our study demonstrated the beneficial effects of FGF18 in alleviating LPS-induced inflammation and preventing endothelial cell leakage in the lung. The overexpression of FGF18 suppressed NF-κB activation, as evidenced by decreased levels of ICAM-1/VCAM-1 and increased levels of VE-cadherin. On the other hand, FGF18 knockdown in mouse models and HUVECs exacerbated endothelial cell leakage. These findings highlight a previously unknown role of FGF18 in regulating the activation of vascular endothelial cells and shed light on the role and mechanism of FGF18 in the pathophysiology of ALI. Consequently, targeting FGF18 may represent a promising therapeutic strategy for the treatment of ALI. It is important to note that FGF18 as a therapeutic agent for ALI would need to be further investigated in preclinical and clinical studies before it can be considered for clinical use.

## Conclusion

In conclusion, these data suggest that FGF18 is associated with ALI and that FGF18 effectively protects against ALI by inhibiting NF-κB mediated by p65 activation. This study enriched the regulatory mechanism of NF-κB in sepsis-associated ALI, suggesting that FGF18 may be a therapeutic pathway for ALI. Considering that inflammation is involved in the pathological processes of many diseases, and that FGF18 effectively inhibits the NF-κB pathway in LPS-induced ALI model, we can further validate its therapeutic potential in many inflammation-related diseases.

### Electronic supplementary material

Below is the link to the electronic supplementary material.

Supplementary Material 1

Supplementary Material 2

## Data Availability

All data generated and analyzed during the study are included in the published article and can be shared upon request.
