# Supplementary material for: FGF18 alleviates sepsis-induced acute lung injury by inhibiting the NF-κB pathway
Source: Respir Res. 2024 Feb 28;25:108. doi: 10.1186/s12931-024-02733-1 (PMC10902988; doi:10.1186/s12931-024-02733-1)
Supplement: Supplementary file 1 — Supplementary Material 1 [file 12931_2024_2733_MOESM1_ESM.docx]

**FGF18 alleviates sepsis-induced acute lung injury by inhibiting the NF-κB pathway**

Zhenyu Hu^1^, Jindan Dai^1^, Tianpeng Xu^1^, Hui Chen^1^, Guoxiu Shen^1^, Jie Zhou^1^, Hongfang Ma^2^, Yang Wang^2*^, Litai Jin^1*^

**
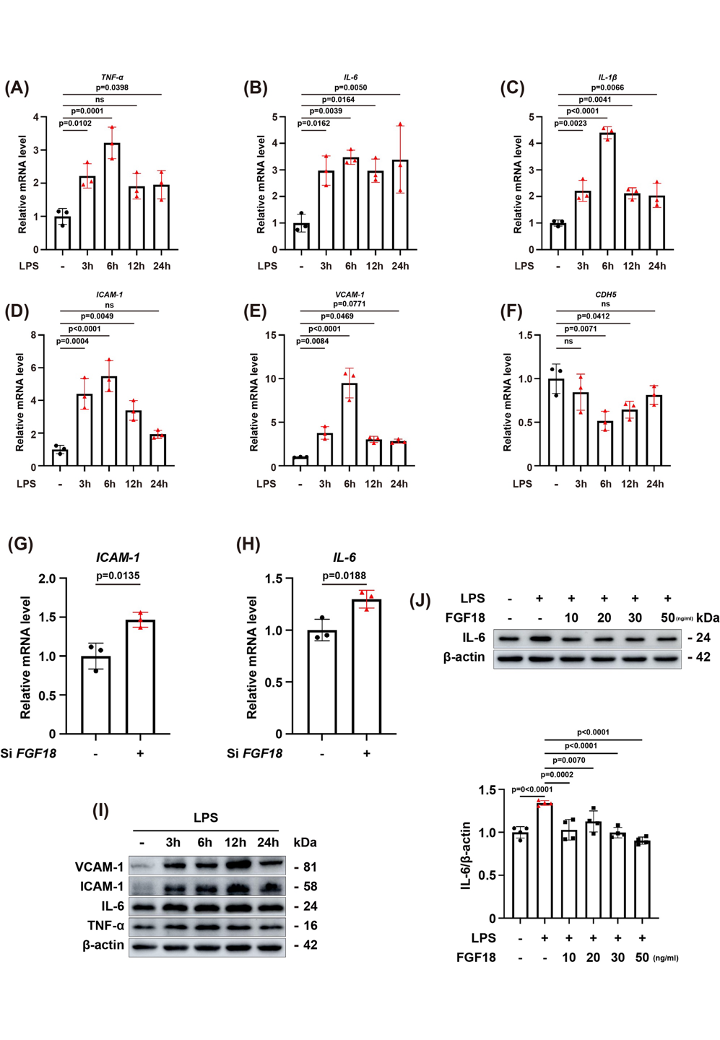
**

**Supplementary Figure 1 Screening of time points and dose points**

(A - F) qRT-PCR analysis of the mRNA levels of TNF-α, IL-6, IL-1β, IACM-1, VCAM-1, and CDH5 in the HUVECs. (n = 3 per group). (G, H) qRT-PCR analysis of the mRNA levels of ICAM-1 and IL-6 in the HUVECs. (n = 3 per group). (I) Western blotting of VCAM-1, ICAM-1, IL-6, and TNF-α in HUVECs were detected. (J) Western blotting of IL-6 in HUVECs was detected. (n = 4 per group).


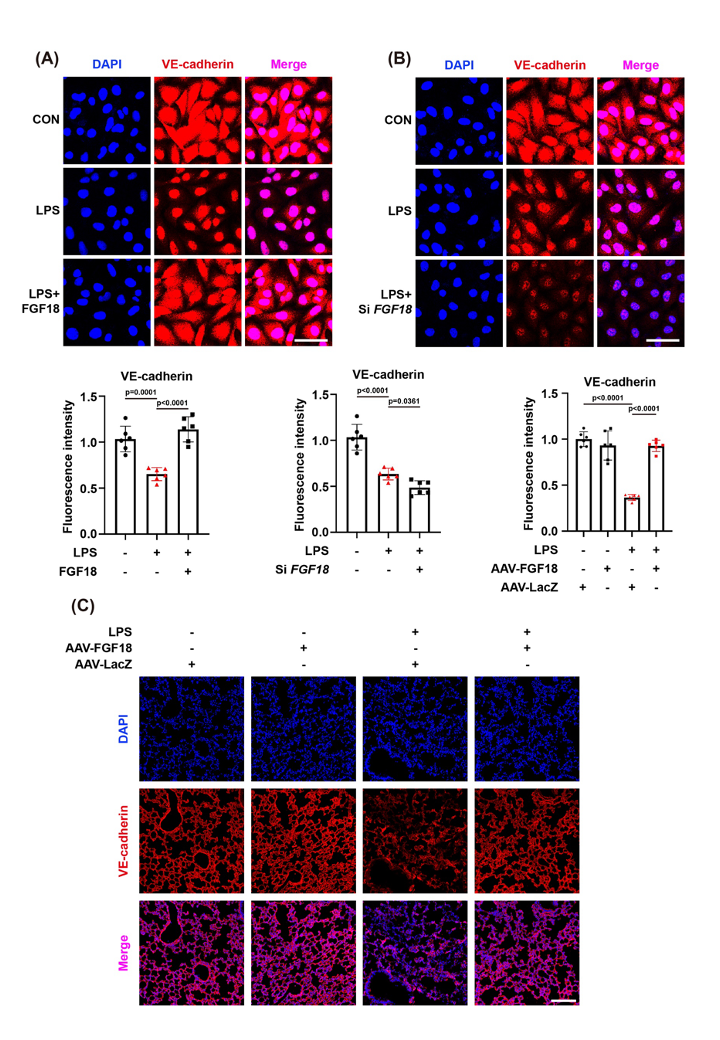


**Supplementary Figure 2** **Immunofluorescent staining of VE-cadherin**

(A, B) Immunofluorescent staining of VE-cadherin (red) and DAPI (blue) in HUVECs were detected. (n = 6 per group, Scale bar = 50 μm). (C) Immunofluorescent staining of VE-cadherin (red) and DAPI (blue) in AAV-FGF18 and AAV-LacZ-treated mice were detected. (n = 6 per group, Scale bar = 150 μm).

**
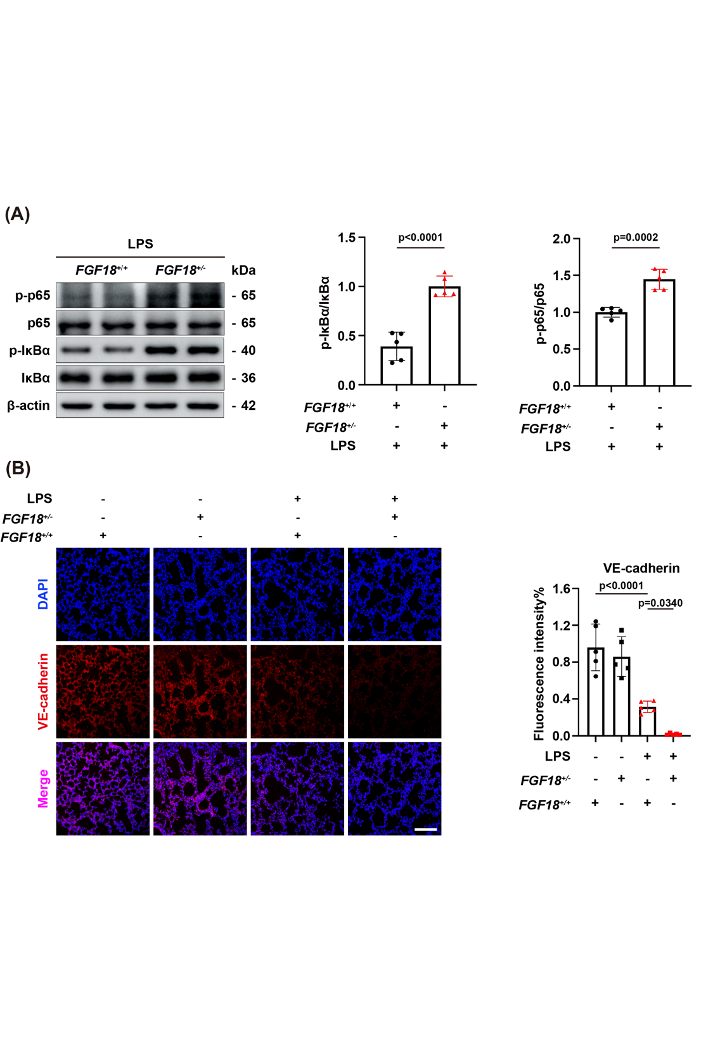
**

**Supplementary Figure 3** **FGF18 deletion exacerbates LPS-induced lung injury**

(A) *FGF18^+/+^* and *FGF18^+/-^* mice were subjected to western blotting analysis. The expression of p-p65, p65, p-IκBα, and IκBα were detected. (n = 5 per group). (B) Immunofluorescent staining of VE-cadherin (red) and DAPI (blue) in *FGF18^+/+^* and *FGF18^+/-^* mice were detected. (n = 5 per group, Scale bar = 150 μm).


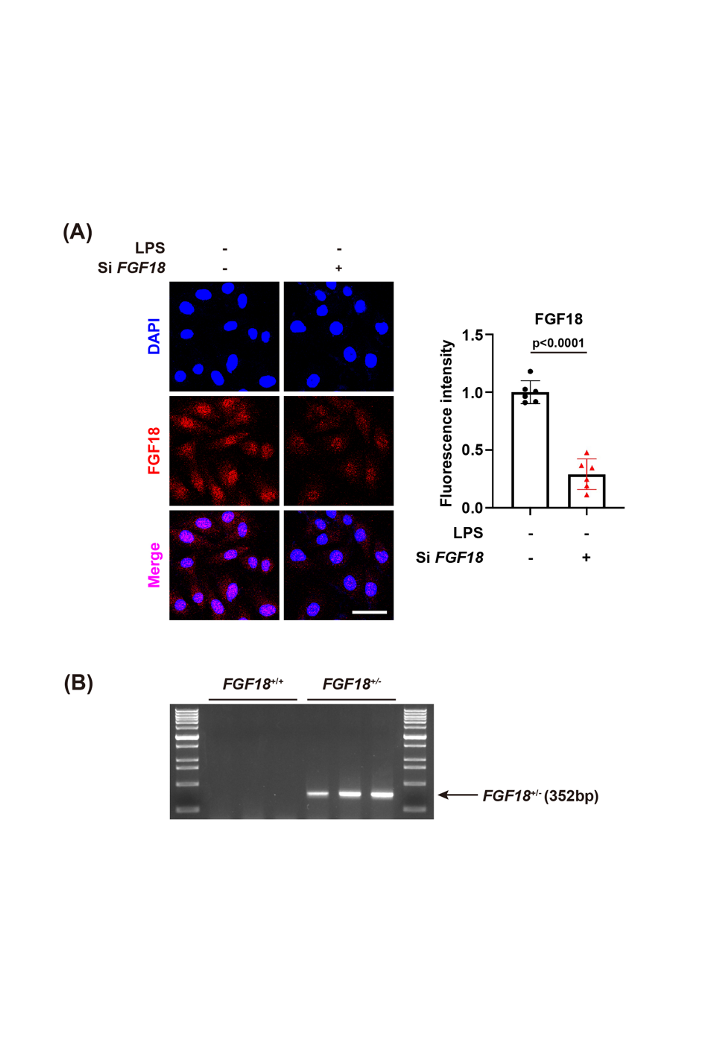


**Supplementary Figure 4** **Reduced expression of FGF18 after gene knockout and si-RNA treatment in vivo and in vitro, respectively**

(A) Immunofluorescent staining of FGF18 (red) and DAPI (blue) in HUVECs were detected. (n = 6 per group, Scale bar = 50 μm). (B) The successful deletion of FGF18 in *FGF18^+/-^* mice was confirmed by RT-PCR.


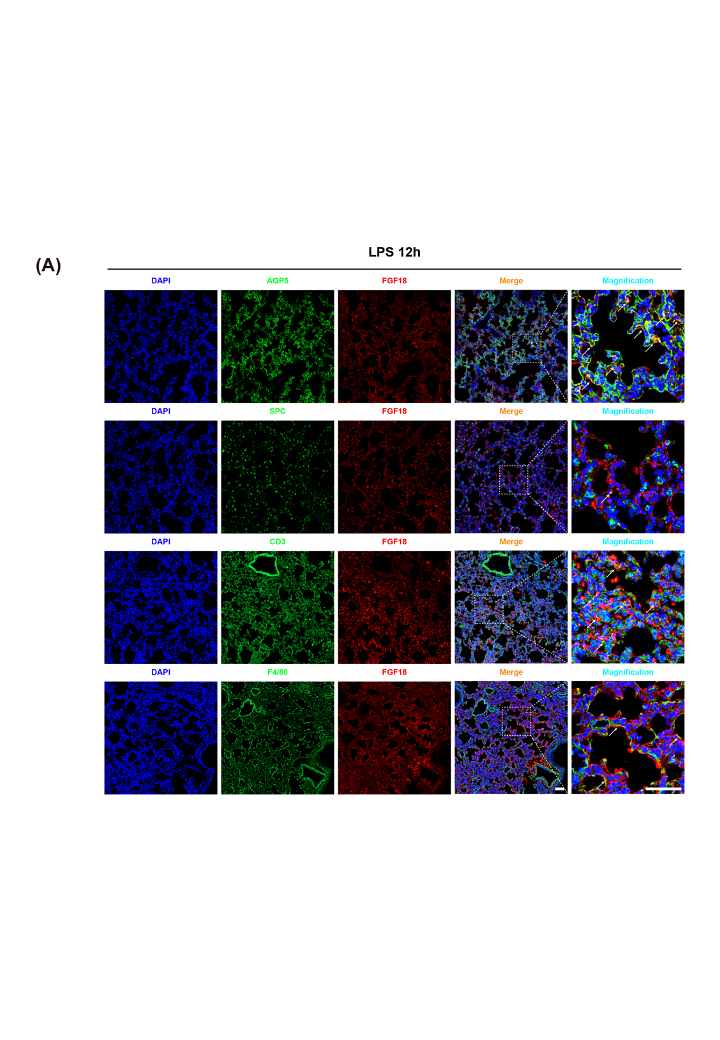


**Supplementary Figure 5 Colocalization of FGF18 and cell Marker**

(A) Immunofluorescent staining of FGF18 (red), Marker (green), and DAPI (blue) in C57BL/6J mice were detected. (Scale bar = 150 μm).


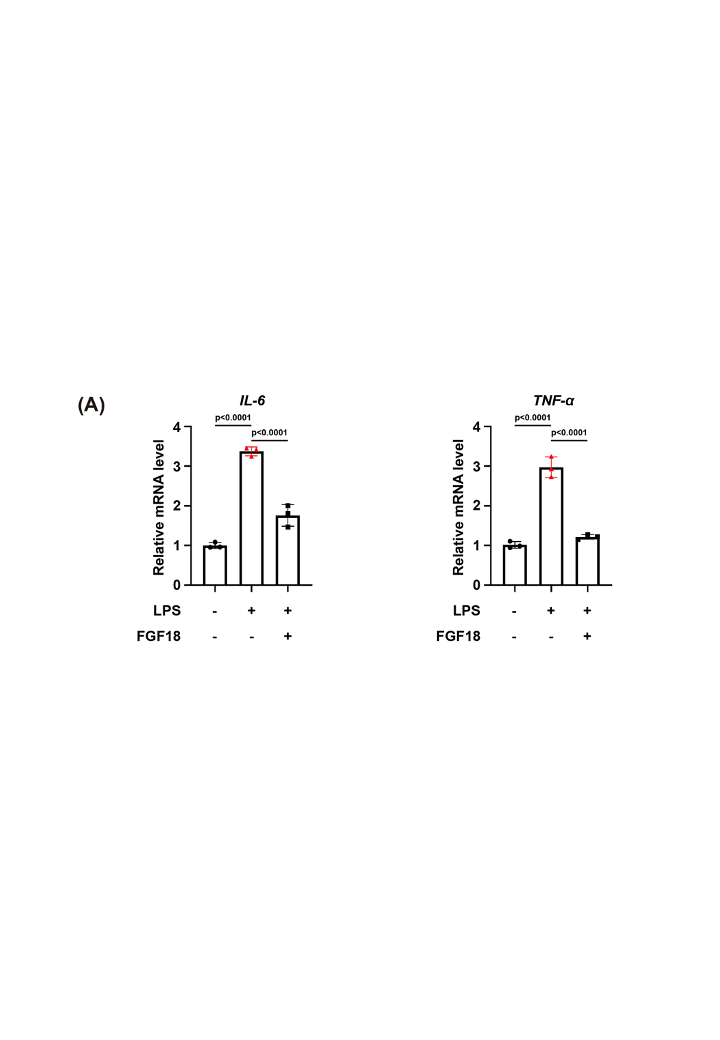


**Supplementary Figure 6** **FGF18 reduces** **mRNA expression of inflammatory factors**

(A) qRT-PCR analysis of the mRNA levels of IL-6 and TNF-α in the HUVECs. (n = 3 per group).


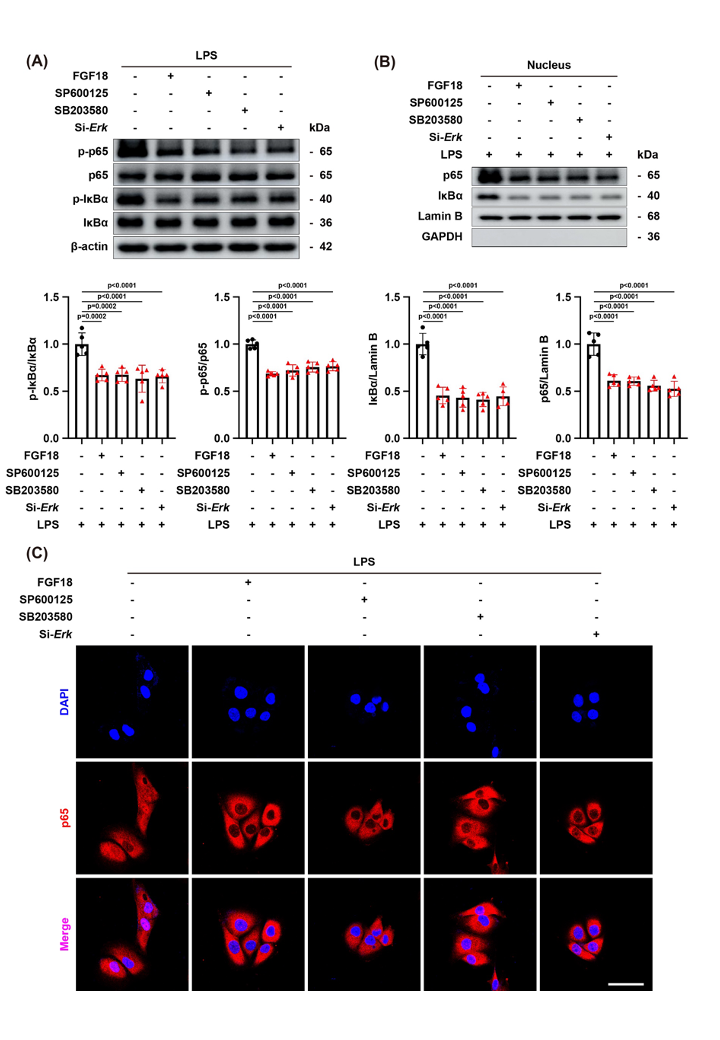


**Supplementary Figure 7 FGF18 inhibits the activation of the NF-κB pathway in HUVECs in line with MAPK kinase inhibitors**

(A) HUVECs were subjected to western blotting analysis. The expression of p-p65, p65, p-IκBα, and IκBα were detected. (n = 5 per group). (B) HUVECs were treated with FGF18 and MAPK kinase inhibitors in the presence of LPS and then subjected to western blotting analysis. Subcellular fractionation of cytoplasm (C) and nuclear fractions (N) from HUVECs. Western blotting analysis for p65, IκBα, GAPDH, and Lamin B. (n = 5 per group). (C) Immunofluorescent staining of p65 (red) and DAPI (blue) in HUVECs were detected. (Scale bar = 50 μm).


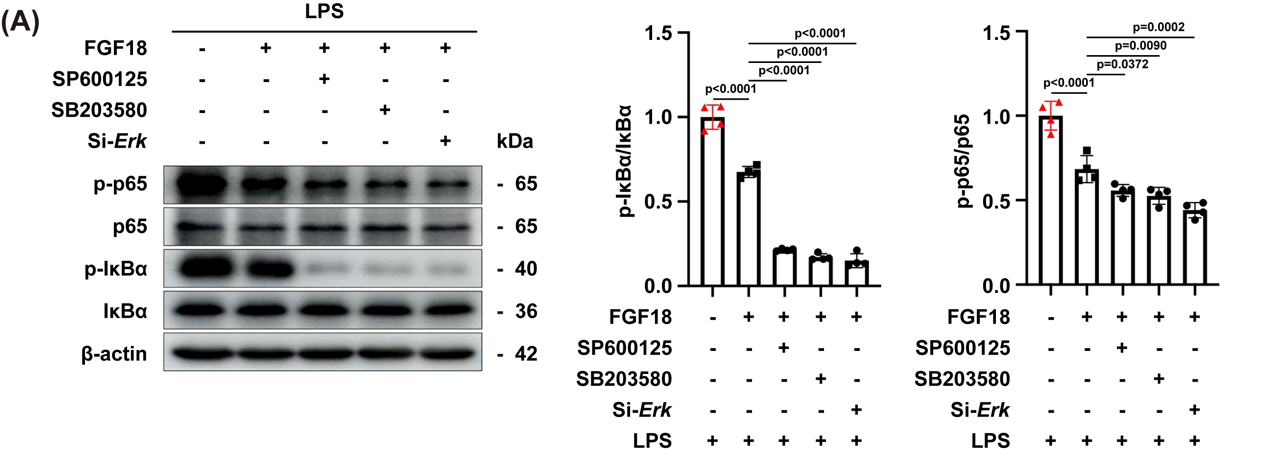


**Supplementary Figure 8 Combined application of FGF18 and MAPK kinase inhibitors further inhibited the NF-κB pathway**

(A) HUVECs were subjected to western blotting analysis. The expression of p-p65, p65, p-IκBα, and IκBα were detected. (n = 4 per group).

**
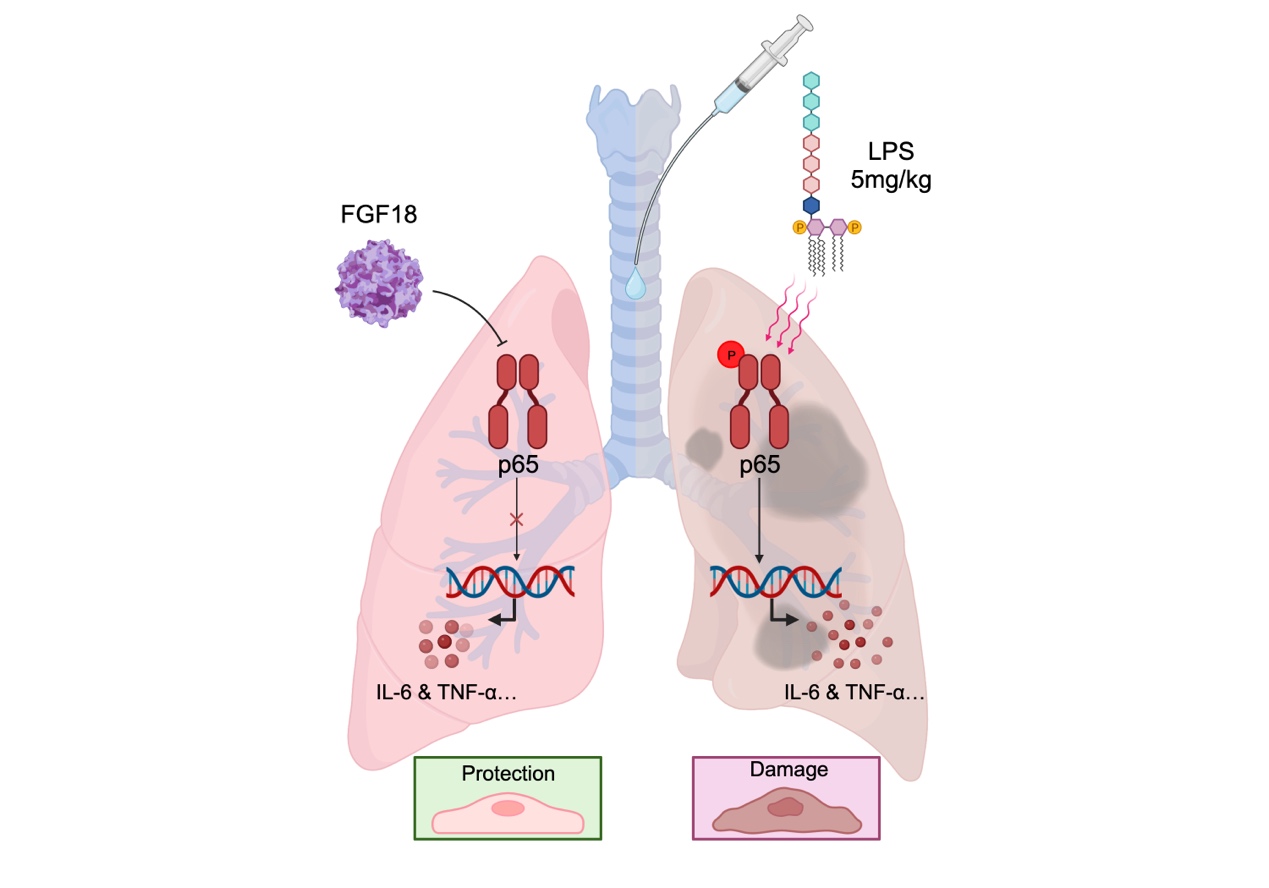
**

**Supplementary Figure 9** **Schematic figure illustrates that FGF18 ameliorates LPS-induced ALI by alleviate NF-κB signaling axis.**

FGF18 negatively regulated NF-κB p65 activity, thereby inhibiting IL-6 and TNF-α generation and protecting lung function by inhibiting endothelial cell damage. In conclusion, the present study identifies a previously unknown biological function of FGF18 in ALI. Created with BioRender.com.

***Supplementary table 1 qRT-PCR and FGF18^+/-^ mice primer sequences***

| Gene Name | Forward Primer | Reverse Primer |
| --- | --- | --- |
| TNF-α | TCCTTCAGACACCCTCAACC | AGGCCCCAGTTTGAATTCTT |
| IL-6 | TGCCAGCCTGCTGACGAAG | AGCTGCGCAGAATGAGATGAG |
| IL-1β | TCTAGGCTTTCAATGAGTGCC | ATCTTTTGGGGTCCGTCAACT |
| ICAM-1 | GGCCGGCCAGCTTATACAC | TAGACACTTGAGCTCGGGCA |
| VCAM-1 | GGGAAGATGGTCGTGATCCTT | TCTGGGGTGGTCTCGATTTTA |
| CDH5 | GGCTCCACAGAGCTCCACTC | TGAGGGATGTTTCTGTTCCGT |
| GAPDH | GACCTGCCGTCTAGAAAAAC | CTGTAGCCAAATTCGTTGTC |
| FGF18^+/−^ | GAAGTGCAAGAACGTGGTGC | CTTTATTAGCCAGAAGTCAGATGC |
