# Supplementary material for: FGF18 alleviates sepsis-induced acute lung injury by inhibiting the NF-κB pathway
Source: Respir Res. 2024 Feb 28;25:108. doi: 10.1186/s12931-024-02733-1 (PMC10902988; doi:10.1186/s12931-024-02733-1)

Fig 1-4

Fig 1

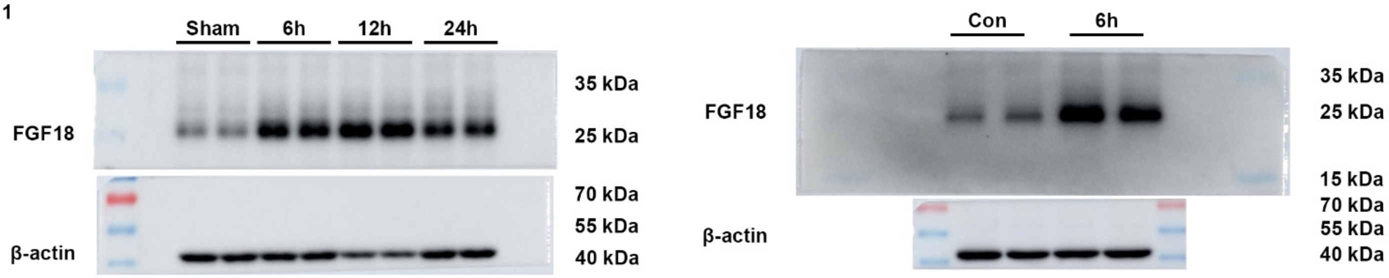

Fig 2

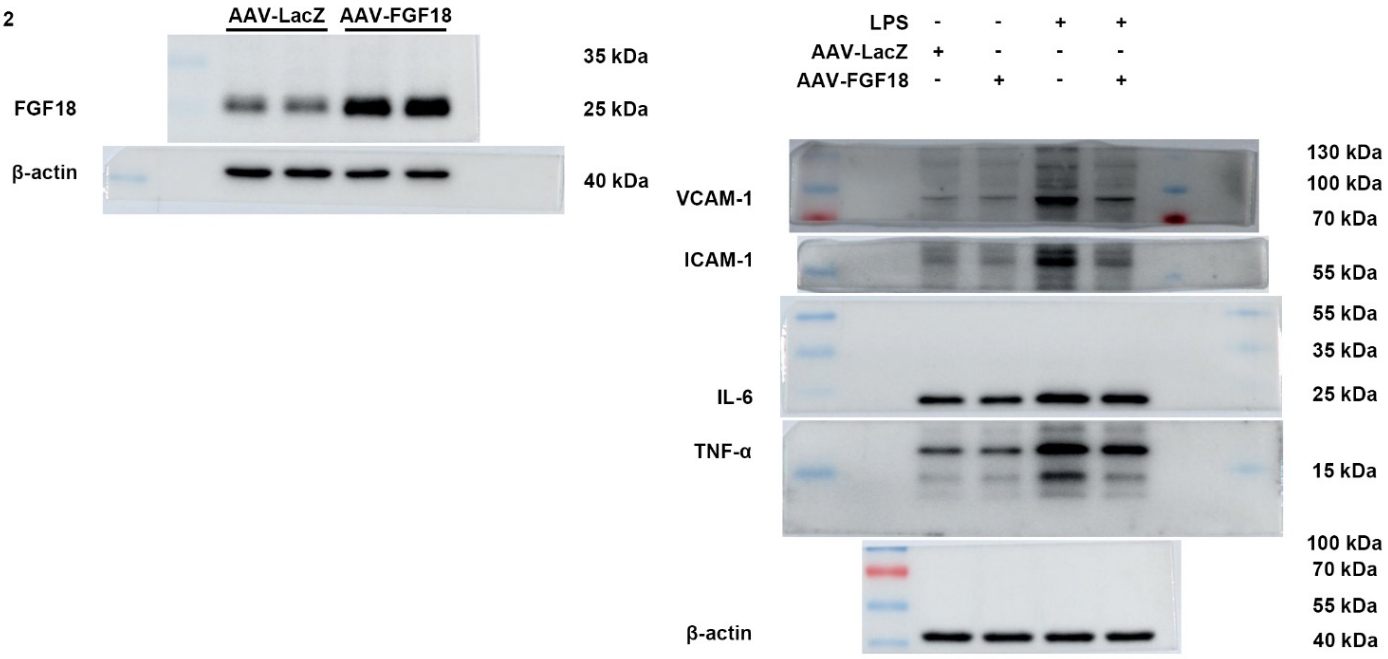

Fig 3

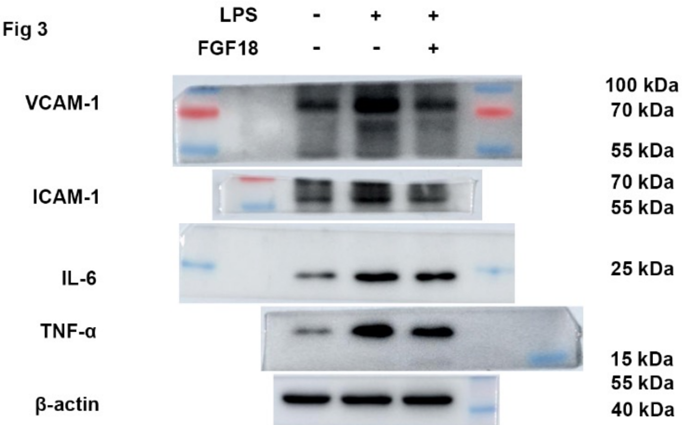

Fig 4

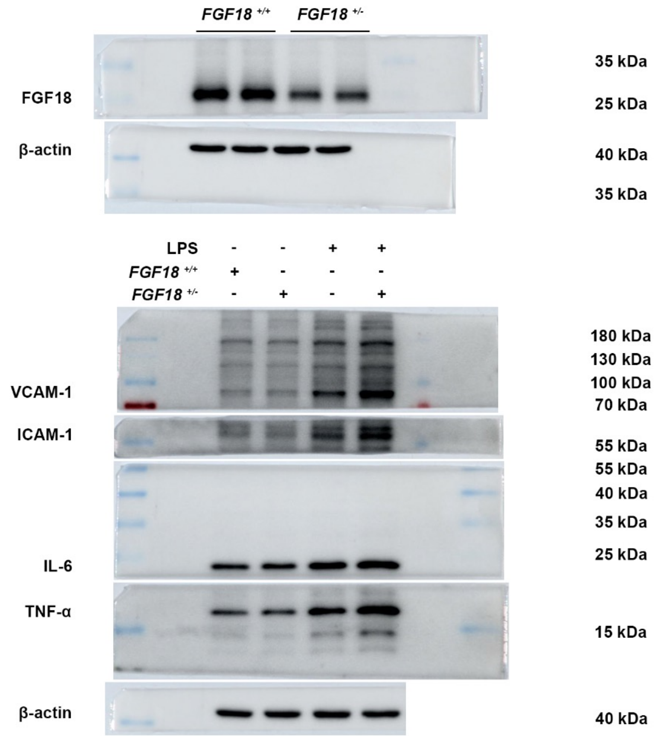

Fig 5-8

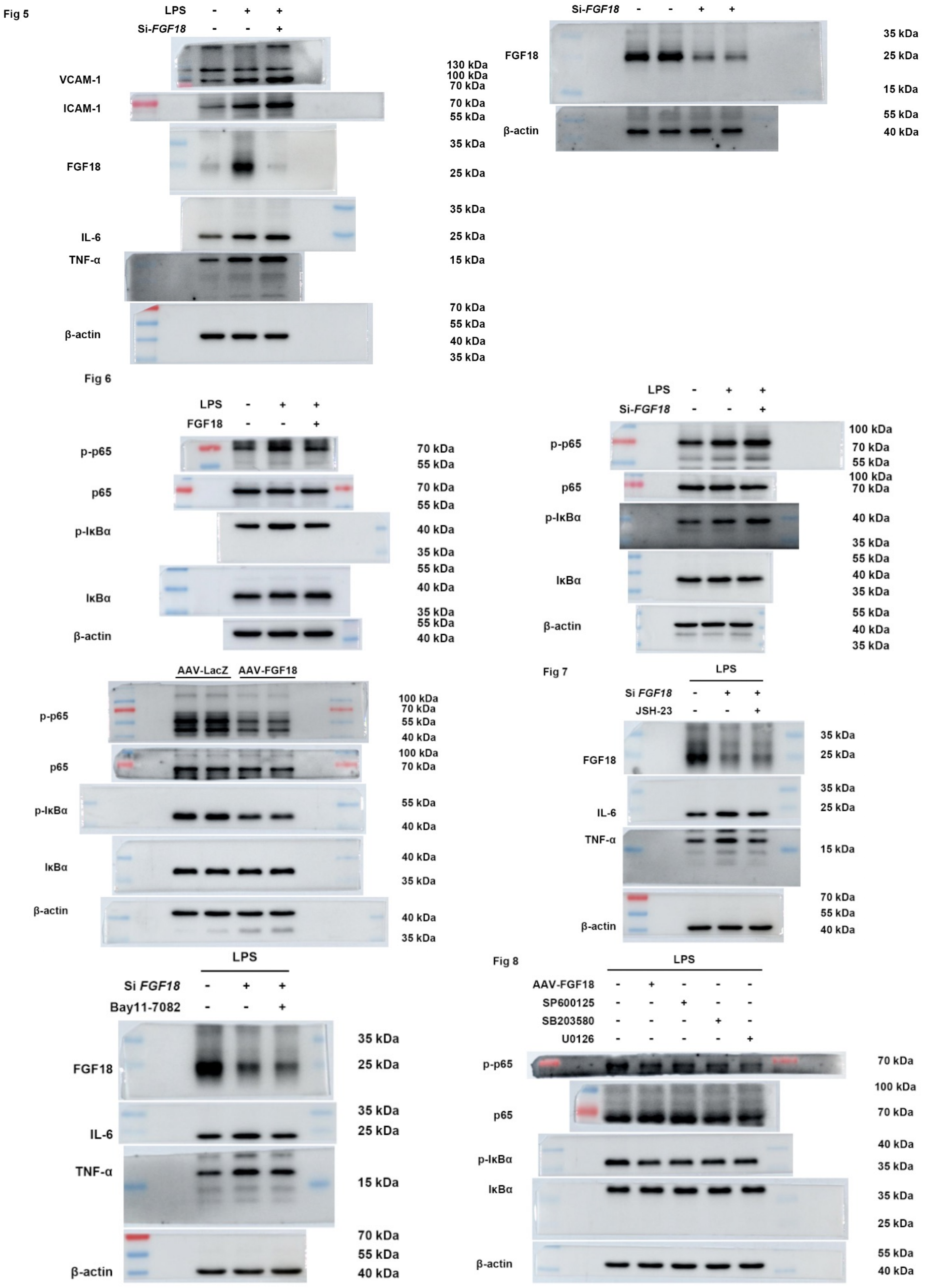

Fig 5
LPS - + +
Si-FGF18 - - +
VCAM-1
ICAM-1
FGF18
IL-6
TNF-α
β-actin
130 kDa
100 kDa
70 kDa
70 kDa
55 kDa
35 kDa
25 kDa
35 kDa
25 kDa
15 kDa
70 kDa
55 kDa
40 kDa
35 kDa
Si-FGF18 - - + +
FGF18
β-actin
35 kDa
25 kDa
15 kDa
55 kDa
40 kDa
Fig 6
LPS - + +
FGF18 - - +
p-p65
p65
p-IκBα
IκBα
β-actin
70 kDa
55 kDa
70 kDa
55 kDa
40 kDa
35 kDa
55 kDa
40 kDa
35 kDa
55 kDa
40 kDa
LPS - + +
Si-FGF18 - - +
p-p65
p65
p-IκBα
IκBα
β-actin
100 kDa
70 kDa
55 kDa
100 kDa
70 kDa
40 kDa
35 kDa
55 kDa
40 kDa
35 kDa
55 kDa
40 kDa
35 kDa
AAV-LacZ
AAV-FGF18
p-p65
p65
p-IκBα
IκBα
β-actin
100 kDa
70 kDa
55 kDa
40 kDa
100 kDa
70 kDa
55 kDa
40 kDa
40 kDa
35 kDa
40 kDa
35 kDa
Fig 7
LPS
Si FGF18 - + +
JSH-23 - - +
FGF18
IL-6
TNF-α
β-actin
35 kDa
25 kDa
35 kDa
25 kDa
15 kDa
70 kDa
55 kDa
40 kDa
LPS
Si FGF18 - + +
Bay11-7082 - - +
FGF18
IL-6
TNF-α
β-actin
35 kDa
25 kDa
35 kDa
25 kDa
15 kDa
70 kDa
55 kDa
40 kDa
Fig 8
LPS
AAV-FGF18 - + - - -
SP600125 - - + - -
SB203580 - - - + -
U0126 - - - - +
p-p65
p65
p-IκBα
IκBα
β-actin
70 kDa
100 kDa
70 kDa
40 kDa
35 kDa
35 kDa
25 kDa
55 kDa
40 kDa

Supplementary Fig 1/3/7/8

Supplementary Fig 1

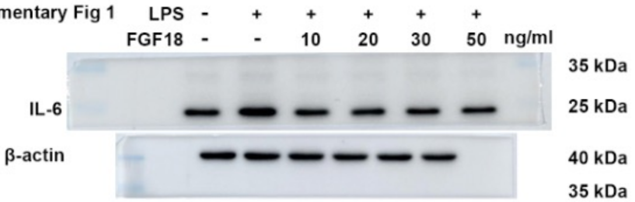

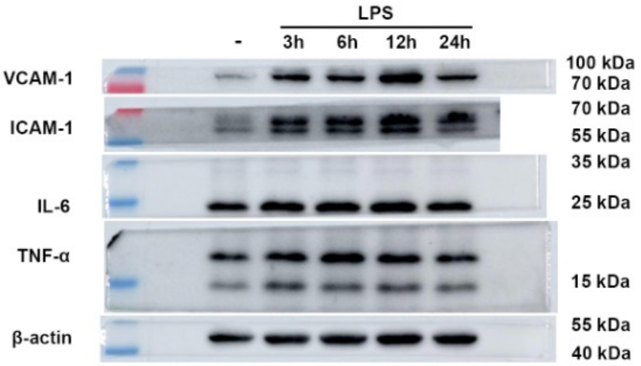

Supplementary Fig 3

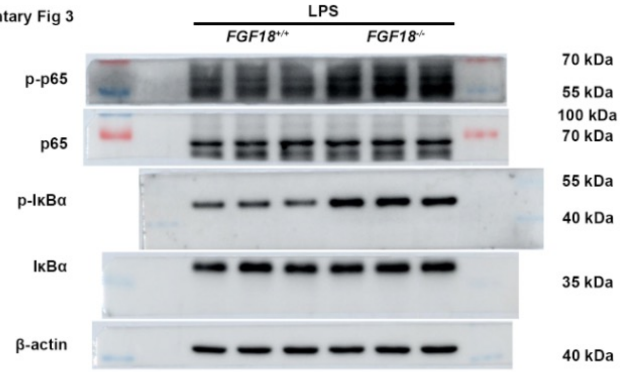

Supplementary Fig 7

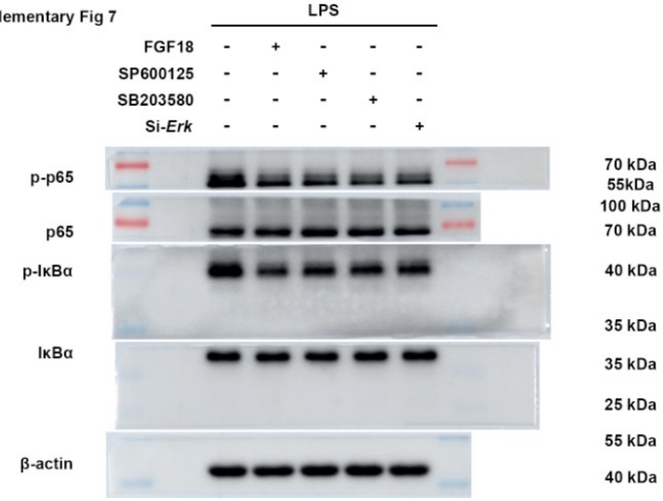

Supplementary Fig 8

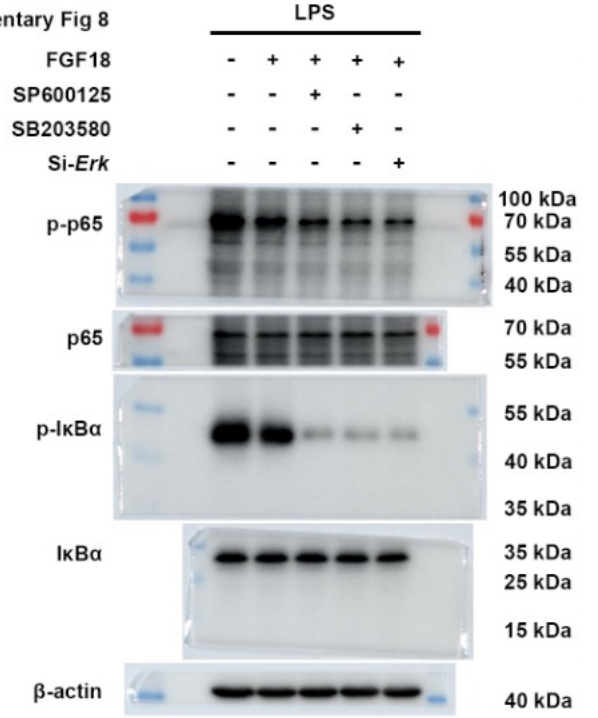

Supplement: Supplementary file 2 — Supplementary Material 2 [file 12931_2024_2733_MOESM2_ESM.pdf]
